# OPA1 and MICOS Regulate mitochondrial crista dynamics and formation

**DOI:** 10.1038/s41419-020-03152-y

**Published:** 2020-10-31

**Authors:** Chao Hu, Li Shu, Xiaoshuai Huang, Jianglong Yu, liuju Li, Longlong Gong, Meigui Yang, Zhida Wu, Zhi Gao, Yungang Zhao, Liangyi Chen, Zhiyin Song

**Affiliations:** 1 Hubei Key Laboratory of Cell Homeostasis, Frontier Science Center for Immunology and Metabolism, College of Life Sciences, Wuhan University, Wuhan, Hubei 430072 China; 2 State Key Laboratory of Membrane Biology, Beijing Key Laboratory of Cardiometabolic Molecular Medicine, Institute of Molecular Medicine, Peking University, Beijing, China; 3 Tianjin Key Laboratory of Exercise Physiology and Sports Medicine, Institute of Sports and Health, Tianjin Sport University, Tianjin, China

**Keywords:** Cell death, Mitochondria

## Abstract

Mitochondrial cristae are the main site for oxidative phosphorylation, which is critical for cellular energy production. Upon different physiological or pathological stresses, mitochondrial cristae undergo remodeling to reprogram mitochondrial function. However, how mitochondrial cristae are formed, maintained, and remolded is still largely unknown due to the technical challenges of tracking mitochondrial crista dynamics in living cells. Here, using live-cell Hessian structured illumination microscopy combined with transmission electron microscopy, focused ion beam/scanning electron microscopy, and three-dimensional tomographic reconstruction, we show, in living cells, that mitochondrial cristae are highly dynamic and undergo morphological changes, including elongation, shortening, fusion, division, and detachment from the mitochondrial inner boundary membrane (IBM). In addition, we find that OPA1, Yme1L, MICOS, and Sam50, along with the newly identified crista regulator ATAD3A, control mitochondrial crista dynamics. Furthermore, we discover two new types of mitochondrial crista in dysfunctional mitochondria, “cut-through crista” and “spherical crista”, which are formed due to incomplete mitochondrial fusion and dysfunction of the MICOS complex. Interestingly, cut-through crista can convert to “lamellar crista”. Overall, we provide a direct link between mitochondrial crista formation and mitochondrial crista dynamics.

## Introduction

Mitochondria contain outer and inner membranes. The inner mitochondrial membrane is organized in two morphologically distinct regions: the inner boundary membrane (IBM) and the crista membrane (CM)^1^. The IBM contains translocases and certain proteins, such as OXA1 and Mia40, to shuttle proteins into the mitochondrial matrix^2^. The CM is usually connected to IBM by the crista junction (CJ), which is a narrow constriction^3,4^. Mitochondrial cristae harbor proteins that establish and maintain their unique structure to provide a large amount of surface area for chemical reactions to occur. Mitochondrial cristae have an orderly arrangement and contain respiratory chain supercomplexes, cytochrome c, and certain proteins that regulate mitochondrial oxidative phosphorylation and electron transfer^3,4^. Three-dimensional (3D) images show that mitochondrial cristae are bag-like structures^1,5^.

Mitochondrial cristae have long been considered tubular or lamellar invaginations formed by protrusion of the IBM into the mitochondrial matrix. OPA1, dimeric F1FO-ATP synthase, and MICOS (mitochondrial contact site and cristae organizing system) complex play key roles during this process^6–8^. OPA1 oligomerization narrows the CJ and promotes ATP synthase dimerization^9,10^. The dimerization of ATP synthase is necessary to control crista biogenesis and morphology, and it induces a positive curvature that results in the formation of a crista sheet at the tip^6^. The MICOS complex regulates the formation of CJs and mediates the contact between the OMM and IMM by interacting with the SAM (the sorting and assembly machinery) complex, which is located on the mitochondrial outer membrane and functions in the assembly of β-barrel proteins into the mitochondrial outer membrane^11,12^. The MICOS complex consists of at least 7 subunits in mammals: Mic60, Mic27, Mic26, Mic25, Mic19, Mic13, and Mic10^3^. Recently, Harner et al. hypothesized that mitochondrial fusion and the MICOS complex contribute to the formation of mitochondrial lamellar cristae^7^.

Under normal conditions, normal tubular or lamellar mitochondrial cristae have an orderly arrangement. However, under different physiological and pathological conditions, the number, size, shape, and distribution of mitochondrial cristae are affected to a very different extent^1,3,4,9,13^. For example, in aged animals, mitochondrial cristae are enlarged, and ~25% of mitochondria contain large central voids that lack CJs^14,15^. However, the mechanism and functions of mitochondrial crista remodeling are largely unclear.

Mitochondria continuously fuse and divide to maintain normal mitochondrial morphology and functions. Mitofusins (Mfn1 and Mfn2) and OPA1 mediate outer and inner mitochondrial membrane fusion, respectively^16,17^. Additionally, Drp1 is a key regulator of mitochondrial fission^18,19^. Theoretically, similar to mitochondria, mitochondrial cristae should be highly dynamic. However, due to the limitation of technology in detecting mitochondrial cristae in living cells, mitochondrial crista dynamics are not fully understood.

For more than half a century, mitochondrial cristae have only been analyzed by transmission electron microscopy (TEM), which cannot be applied to living cells. Recently, the development of super-resolution microscopy technology has provided an excellent tool for visualizing mitochondria or mitochondrial cristae in living cells^20–26^. We previously established a super-resolution microscopy technology called structured illumination microscopy (Hessian-SIM), which enables fast, long-term, and super-resolution imaging of mitochondrial cristae^27^. Here, we used Hessian-SIM combined with TEM, focused ion beam/scanning electron microscopy (FIB-SEM), and 3D tomographic reconstruction to analyze the dynamic changes in mitochondrial cristae and to study the role of the related factors in regulating mitochondrial crista shape, quantity, arrangement, and distribution.

## Results

### Mitochondrial cristae are highly dynamic and communicate with each other

**Fig. 1:**
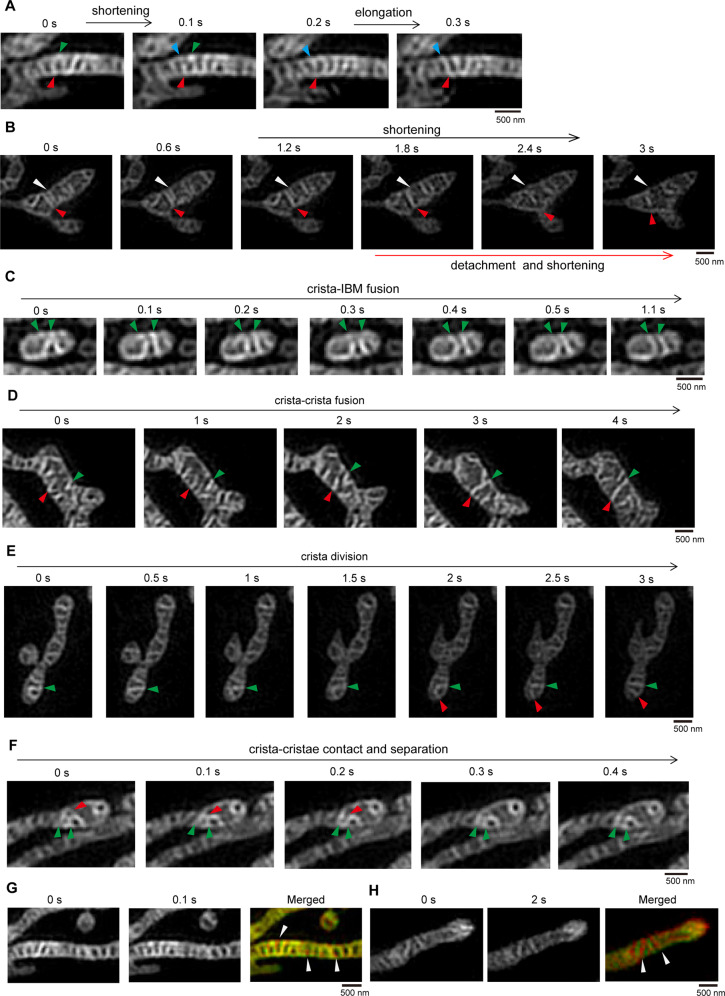
Mitochondrial cristae dynamics in live cells. **A** Representative live-cell time-lapse images of mitochondria in HeLa cells. Mitochondria in HeLa cells were stained with MitoTracker Green (250 nM, 15 min), then were tracked by time-lapse Hessian-SIM (90 frames/sec), 9 raw frames were used to reconstruct one super-resolution image. The indicated images demonstrate the mitochondrial cristae can shorten or extension in a very short period of time. Green, red, and blue arrowheads indicate the dynamic cristae. **B** Representative live-cell time-lapse images of mitochondrial cristae detached from the inner boundary membrane (IBM) and subsequently shortening. HeLa cells were treated with MitoTracker Green (250 nM, 15 min) and tracked by time-lapse by Hessian-SIM as described in (**A**). The white arrows marked the continuous shortening of mitochondria cristae, respectively, the red arrows indicate the cristae detached from the mitochondrial IBM then shorten in the mitochondrial matrix. **C** Representative live-cell time-lapse images of mitochondrial cristae fusing with IBM. HeLa cells were treated with MitoTracker Green (250 nM, 15 min) and tracked by time-lapse Hessian-SIM as described in (**A**). The green arrowheads showed that the site of mitochondrial cristae fusing with the IBM. **D** Representative live-cell Hessian-SIM images of mitochondrial cristae fusion event. HeLa cells were treated with MitoTracker Green (250 nM, 15 min) and tracked by time-lapse Hessian-SIM as described in (**A**). The selected images show that two mitochondrial cristae fused into one crista, the green and red arrows mark the mitochondrial cristae fusion event. **E** Representative live-cell Hessian-SIM images of mitochondrial crista division event. HeLa cells were stained with MitoTracker Green (250 nM, 15 min) and tracked by time-lapse Hessian-SIM as described in (**A**). The green and red arrows indicate the mitochondrial crista division process within 3 s. **F** Representative live-cell time-lapse images of mitochondrial crista–crista contact. HeLa cells were treated with MitoTracker Green (250 nM, 15 min) and tracked by time-lapse Hessian-SIM as described in (**A**). The green and red arrowheads-indicated mitochondrial cristae contacted each other and then were separated. **G**, **H** HeLa cells were stained with MitoTracker Green (250 nM, 15 min) and tracked by time-lapse Hessian-SIM as described in (**A**). The selected two images (*t* = 0 s, 0.1 s) (**G**) or (*t* = 0 s, 2 s) (**H**) were merged to show mitochondrial crista dynamic change. The white arrowheads indicate the moved mitochondrial cristae.

Hessian-SIM allows MitoTracker Green FM (specifically stains mitochondria independent of mitochondrial membrane potential)-labeled mitochondrial cristae to be visualized and tracked in living cells^27^. We used Hessian-SIM to study the dynamic changes in mitochondrial cristae in living cells. We found that the membrane components of the mitochondrial IBM and cristae membrane were not evenly distributed within the mitochondria (Figs. S1A–S1C). Therefore, communication and material exchange between the mitochondrial cristae and IBM should occur to maintain mitochondrial membrane homeostasis. In living cells, mitochondrial cristae were mainly tubular or lamellar, highly dynamic, and organized, with remarkable changes in length and position within one second (Fig. 1A–C and Supplementary Video 1). The mitochondrial cristae constantly altered their lengths by elongation or shortening, detached from the IBM, or contacted and fused with the IBM (Fig. 1A–C and S1D), suggesting that mitochondrial cristae are dynamically altered in length and constantly contact and communicate with the IBM. Moreover, two mitochondrial cristae could fuse into one (Fig. 1D), and one crista could divide into two (Fig. 1E). Occasionally, mitochondrial cristae contacted each other and fused to form a crista network; however, this contact was subsequently separated (Fig. 1F), suggesting that mitochondrial cristae contact each other via a “kiss-and-run” mode. We also measured the mitochondrial crista movement rate and found that mitochondrial IBMs had a substantial overlap, but a few mitochondrial cristae did not, as seen by comparing the 0-sec and 0.1-sec images (Fig. 1G). However, most mitochondrial cristae showed no overlap between the 0-sec and 2-sec images (Fig. 1H). Additionally, mitochondrial cristae can undergo elongation, shortening, detachment, fusion, and fission in one or a few seconds (Fig. 1A–F). These data suggest that mitochondrial cristae are highly dynamic and change within a few seconds.

**Fig. 2:**
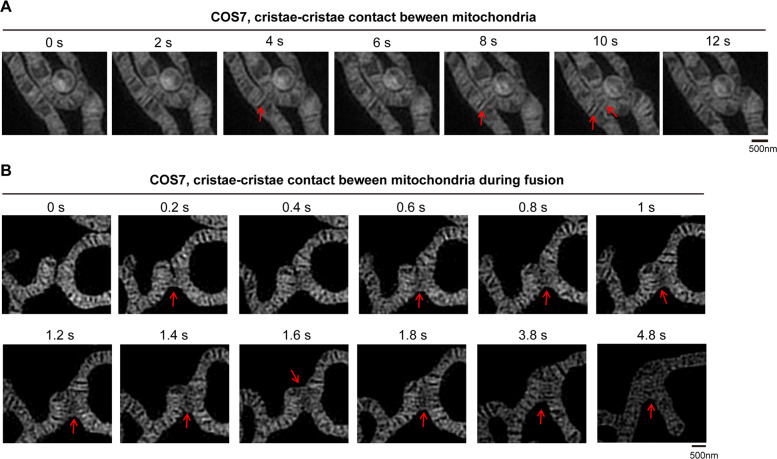
Mitochondrial crista–crista contact between two mitochondria. **A**, **B** Representative live-cell time-lapse Hessian-SIM images of mitochondria in HeLa (**A**) or COS7 (**B**) cells stained with MitoTracker Green (250 nM, 15 min). Time-lapse Hessian-SIM images reveal that mitochondrial crista–crista contact between two mitochondria during mitochondrial fusion. The red arrows demonstrate crista–crista contact.

Mitochondria can contact each other by a “kiss-and-run” mode^28^. We then investigated whether mitochondrial cristae are involved in mitochondrion–mitochondrion contact by Hessian-SIM. During mitochondrion–mitochondrion contact (kiss-and-run fusion), direct mitochondrial crista–crista contact was observed (Fig. 2A). Moreover, during mitochondrial fusion, mitochondrial cristae contact and fuse with each other (Fig. 2B). Our data are consistent with a previous report indicating that the cristae of adjacent mitochondria are coordinated at inter-mitochondrial junctions in cardiomyocytes^29^. Overall, these findings suggest that mitochondrial cristae contact and communicate with each other within and between mitochondria.

**Fig. 3:**
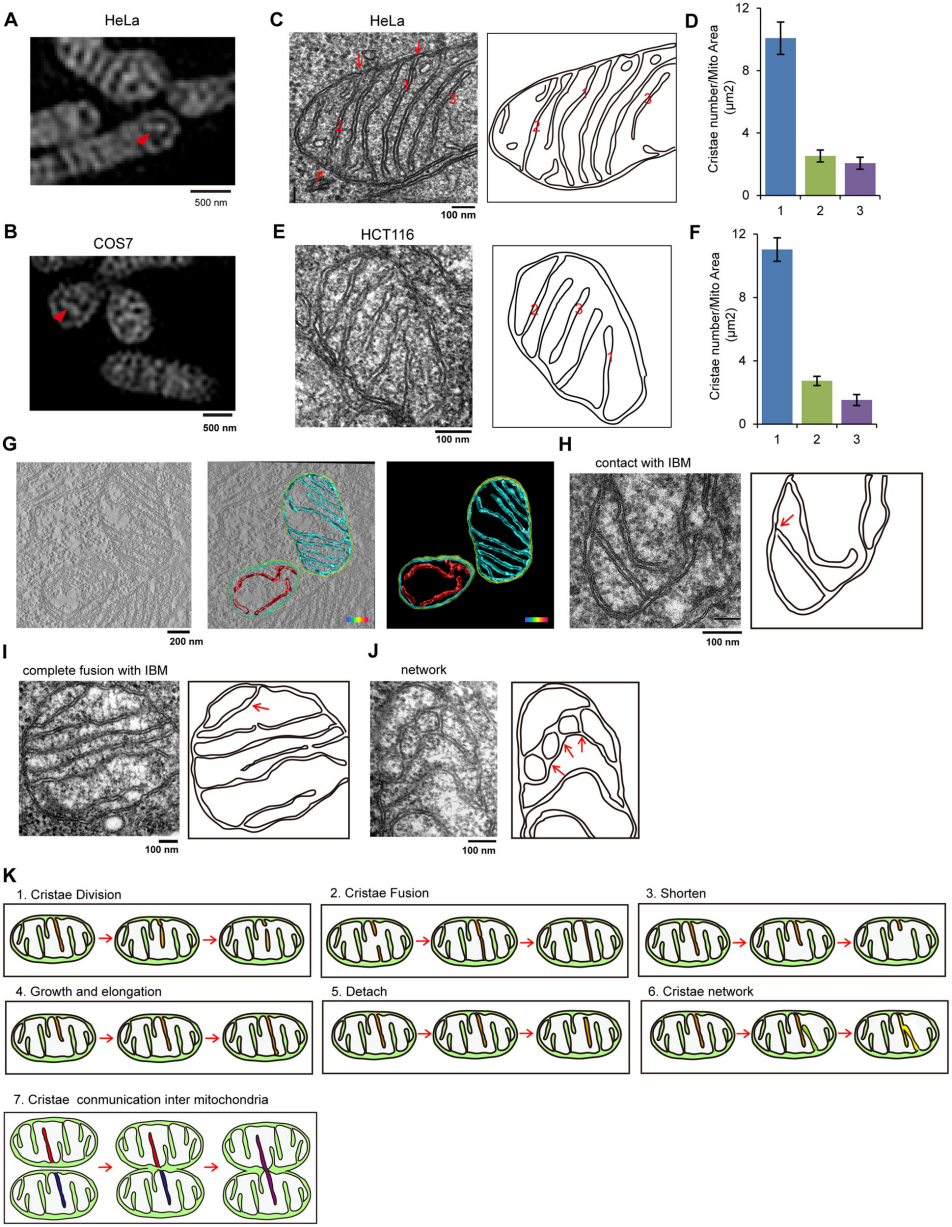
Mitochondrial cristae types and dynamic events. **A**, **B** HeLa (**A**) and COS-7 (**B**) cells were stained with MitoTracker Green (250 nM, 15 min), and then imaged by Hessian-SIM. The white arrows marked the mitochondrial cristae lacking crista junction. **C**–**F** Mitochondrial cristae in HeLa (**C**) or HCT116 (**E**) cells were analyzed and imaged by transmission electron microscopy (TEM), and the representative image of mitochondrial ultrastructure was drawn. The red numbers represent the different types of mitochondrial cristae morphology. “1” represents mitochondrial crista with one crista junction (CJ), “2” represents mitochondrial crista containing two CJs, “3” represents mitochondrial crista lacking CJs. Then, the number of HeLa (**D**) or HCT116 (**F**) mitochondrial cristae types were counted (three independent experiments, 100 mitochondria cristae for each experiment). **G** HeLa cells were fixed, filtrated, polymerized, and embedded in EPON 812 resin, the specimen of 70 nm sections was then imaged using Fei Tecnai Spirit Transmission Microscope. There-dimensional (3D) reconstruction and segmentation of mitochondria were completed using 3D-IMOD software. Cyan: mitochondrial crista containing CJ; red, mitochondrial crista lacking CJ. **H**–**J** HeLa mitochondrial cristae were analyzed and imaged by TEM, and the representative images of mitochondrial ultrastructure were drawn. The indicated mitochondrial cristae dynamic events were displayed, the red arrows in the drawing picture indicate the cristae contacting IBM (**H**), crista containing two CJs (**I**), or crista–crista contact site (**J**). **K** The models of mitochondrial cristae dynamics. 1. Mitochondrial cristae division: one crista splits into two independent cristae; 2. Mitochondrial cristae fusion: two cristae fused into one crista; 3. Mitochondrial cristae shorten: the length of cristae is shortened; 4. Mitochondrial growth and elongation: crista undergoes growth and elongation; 5. Mitochondrial crista detachment: crista is detached from IBM, meanwhile, lost a Cj; 6. Mitochondrial cristae network: cristae can fuse with each other to form a network structure. 7. Mitochondrial cristae communication inter-mitochondria: cristae contacts and communicates with each other between two mitochondria.

Due to the limitation of Hessian-SIM resolution, we could not distinguish crista–IBM and crista-cristae fusion or contact; thus, we investigated mitochondrial crista morphology by TEM. The contact site between mitochondrial cristae and the IBM is called the CJ. Based on the number of CJs per crista, mitochondrial crista could be classified into 3 categories: (1) type I cristae, mitochondrial crista containing one CJ; (2) type II cristae, mitochondrial crista containing two CJs; and (3) type III cristae, mitochondrial crista without CJ. Most mitochondrial cristae of HeLa or COS7 cells belong to types I and II, and only a few cristae are type III (Fig. 3A, B). Under normal conditions, the mitochondrial cristae are mainly type I, and a few type II and type III cristae exist in HeLa and HCT116 cells (Fig. 3C–F). Consistently, 3D tomographic reconstruction of mitochondria further confirmed the existence of type I, II, and III mitochondrial cristae (Fig. 3G). The prevailing view is that the IBM can invaginate into the mitochondrial matrix to form mitochondrial cristae^6,30^, and this theory explains the existence of type I mitochondrial cristae. However, the existence of type III cristae indicates that mitochondrial cristae are able to detach from the IBM. The type II cristae, on the other hand, suggests that mitochondrial cristae can fuse with the IBM. Then, we analyzed a large number of mitochondria and captured TEM images displaying contact and fusion between cristae and the IBM (Fig. 3H, I) or images representing the crista network (Fig. 3J). These data further support the dynamic characteristics of mitochondrial cristae.

Based on our findings, we propose a new understanding of mitochondrial crista dynamics (Fig. 3K). In living cells, mitochondrial cristae are tubular and lamellar and highly ordered in mitochondria; they are highly dynamic and continuously elongated or shortened. Importantly, mitochondrial crista dynamics are highly associated with crista biogenesis: crista elongation is related to crista formation, crista–IBM contact and fusion can form CJs, and detachment of crista from the IBM leads to the loss of CJs.

### OPA1 and Yme1L regulate mitochondrial crista formation, morphology, arrangement, and dynamics

**Fig. 4:**
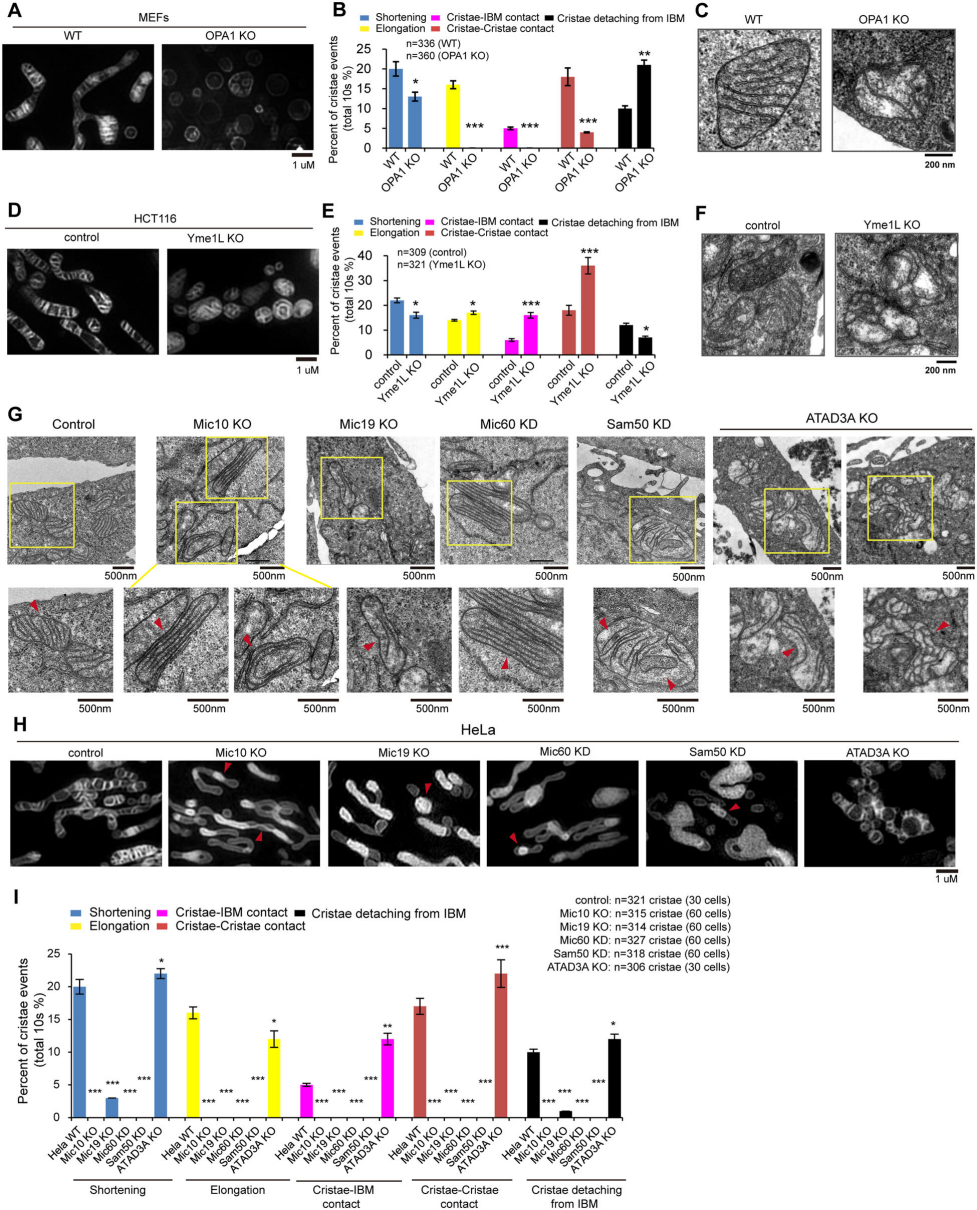
Mitochondrial cristae dynamics is regulated by OPA1, Yme1L, MICOS complex, and ATAD3A. **A** Representative Hessian-SIM images of mitochondria in Live WT and OPA1 knockout (KO) MEFs stained with MitoTracker Green (250 nM, 15 min). **B** Live WT and OPA1 KO MEFs were stained with MitoTracker Green (250 nM, 15 min), and tracked and imaged by time-laps Hessian-SIM. Mitochondrial cristae dynamic events, including shortening, elongation, crista–IBM contact, crista–crista contact, and crista detaching from IBM were quantified (three independent experiments, about 100 mitochondria cristae for each experiment, total mitochondria cristae were indicated). Statistical significance was assessed from the student’s *t*-test; error bars indicate the means ± SD of three independent experiments, N.S. indicates “not significant”, ***p < 0.001 versus control. **C** Representative TEM images of mitochondria in WT and OPA1 KO MEFs. **D** Representative Hessian-SIM images of mitochondria in live control and Yme1L KO HCT116 cells stained with MitoTracker Green (250 nM, 15 min). **E** Live control and Yme1L KO HCT116 cells were stained with MitoTracker Green (250 nM, 15 min), and tracked and imaged by time-laps Hessian-SIM. Mitochondrial cristae dynamic events, including shortening, elongation, crista–IBM contact, crista–crista contact, and crista detaching from IBM were quantified (three independent experiments, about 100 mitochondria cristae for each experiment, total mitochondria cristae were indicated). Statistical significance was assessed from the student’s *t*-test; error bars indicate the means ± SD of three independent experiments, N.S. indicates “not significant”, *p < 0.05, ***p < 0.001 versus control. **F** Representative TEM images of mitochondria in control and Yme1L KO HCT116 cells. **G** Representative TEM images of mitochondria in control, Mic10 KO, Mic19 KO, Mic60 knockdown (KD), Sam50 KD, and ATAD3A KO HeLa cells. Magnified images in the boxed regions are shown as insets. **H** Live control, Mic10 KO, Mic19 KO, Mic60 KD, Sam50 KD, and ATAD3A KO HeLa cells were stained with MitoTracker Green (250 nM, 15 min) and imaged by Hessian-SIM. The representative mitochondrial cristae were displayed. **I** Live control, Mic10 KO, Mic19 KO, Mic60 KD, Sam50 KD, and ATAD3A KO HeLa cells were stained with MitoTracker Green (250 nM, 15 min), and tracked and imaged by time-laps Hessian-SIM. Mitochondrial cristae dynamic events, including shortening, elongation, crista–IBM contact, crista–crista contact, and crista detaching from IBM were quantified (three independent experiments, about 100 mitochondria cristae for each experiment, total mitochondria cristae were indicated). Statistical significance was assessed from the student’s t-test; error bars indicate the means ± SD of three independent experiments, **p < 0.01, ***p < 0.001 versus control.

OPA1 is required for maintaining mitochondrial cristae^31^. We thus investigated the role of OPA1 in mitochondrial crista dynamics. In WT MEFs, most of the mitochondrial cristae were straight, had an orderly distribution in the mitochondria and were nearly parallel to each other (Fig. 4A). Importantly, WT MEF mitochondrial cristae were highly dynamic, displaying shortening, elongation, contact with IBM, etc. (Fig. 4B and Supplementary Video 2). In OPA1 knockout (KO) MEFs, mitochondria were fragmented, mitochondrial cristae were decreased, and crista dynamics, including shortening, elongation, crista–IBM contact (or fusion), and crista–crista contact (or fusion), were markedly decreased (Fig. 4A, B Supplementary Video 3), indicating that OPA1 regulates mitochondrial crista formation and dynamics. Interestingly, crista detachment from the IBM (loss of CJs) was increased in OPA1 KO mitochondria (Fig. 4A, B and Supplementary Video 3), suggesting that OPA1 is associated with the maintenance of CJs. Our findings are consistent with a previous report that OPA1 oligomerization is involved in the maintenance of CJs^32^, indicating that OPA1 may form a “staple” across CJs. Consistently, TEM imaging confirmed that OPA1 KO led to decreased mitochondrial cristae and caused a relative increase in abnormal mitochondrial cristae that are crooked, are disordered, or contain 0 or 2 crista junctions (Fig. 4C and S2A–S2C). Moreover, OPA1 KO mitochondria displayed relatively increased CJs per crista and increased crooked cristae compared with WT mitochondria (Fig. 4C and S2D–S2E). Interestingly, type II cristae are relatively stable, displaying few crista shortening and elongation events (Supplementary Video 3).

Yme1L is an inner mitochondrial membrane protease that cleaves OPA1 at the S2 site, and Yme1L depletion results in increased OMA1-mediated OPA1 processing at the S1 site^33–36^. Therefore, we investigated whether Yme1L regulates mitochondrial crista dynamics. Hessian-SIM and TEM imaging revealed that Yme1L KO caused mitochondrial fragmentation and led to fewer mitochondrial cristae, more abnormal mitochondrial cristae, and more CJs per crista (Fig. 4D–F and S2F–S2I). Furthermore, most Yme1L KO mitochondrial cristae were crooked and arranged in a disordered manner (Fig. 4F and S2J). In addition, Yme1L KO mitochondrial cristae, which were abnormal in shape, were also highly dynamic and moved quickly, similar to the control mitochondrial cristae, but the crista dynamic events of shortening and detachment from the IBM were markedly decreased (Fig. 4E and Supplementary Videos 4 and 5), suggesting that Yme1L is an important regulator of mitochondrial crista dynamics. Interestingly, crista–IBM and crista–crista contact (or fusion) events were remarkably increased in Yme1L KO mitochondria compared with the control (Fig. 4E and Supplementary Videos 5), suggesting that Yme1L regulates mitochondrial crista–crista communication.

### The MICOS complex and its associated proteins regulate mitochondrial crista junction formation, crista–IBM fusion, and cristae distribution

We assessed the role of MICOS complex subunits, including Mic10, Mic19, and Mic60, in the regulation of mitochondrial CJs. As seen by TEM analysis, Mic10 KO, Mic19 KO, or Mic60 knockdown (KD) resulted in a slight decrease in the number of mitochondrial cristae, an increase in abnormal cristae, and a complete loss of mitochondrial CJs (Fig. 4G and S2K–S2P). Furthermore, a large number of mitochondrial cristae were distributed parallel to the IBM, and a few mitochondrial cristae exhibited an onion-like structure in Mic10 KO, Mic19 KO, and Mic60 KD cells (Fig. 4G). Further time-lapse Hessian-SIM showed that mitochondrial cristae were unevenly distributed in the mitochondria in Mic10 KO, Mic19 KO and Mic60 KD cells, and the fluorescence of cristae was stronger than that of the IBM (Fig. 4H). Moreover, in Mic10 KO, Mic19 KO, and Mic60 KD cells, mitochondrial crista dynamic events, including shortening, elongation, and crista–crista contact (or fusion), were remarkably decreased (Fig. 4I and Supplementary Videos 6–8), suggesting that the MICOS complex regulates mitochondrial crista dynamics. Additionally, the fluorescence of Mic10 KO, Mic19 KO, and Mic60 KD cristae did not diffuse into the IBM (Fig. 4H and Supplementary Videos 6–8), indicating that Mic10 KO, Mic19 KO, and Mic60 KD cristae do not fuse with the IBM due to the loss of CJs. These results suggest that the MICOS complex is essential for mitochondrial crista junction formation and crista–IBM fusion. Additionally, only a few mitochondrial cristae were observed by Hessian-SIM (Fig. 4H), although many cristae exist in Mic10 KO, Mic19 KO, and Mic60 KD mitochondria (Fig. 4G), probably due to changes in the mitochondrial membrane organization or the composition of the inner mitochondrial membrane, which leads to little staining with MitoTrack Green^22^.

The Sam50-Mic19-Mic60 axis plays a key role in mitochondrial crista junction formation^37^. Hessian-SIM showed that Sam50-GFP signals were puncta that faced CJs (Fig. S2Q–S2R). Thus, we investigated the effect of Sam50 KD on mitochondrial cristae. Sam50 KD reduced the number of mitochondrial cristae and resulted in a significant decrease in the number of CJs per mitochondrial crista but an increased number of abnormal mitochondrial cristae (Fig. 4G and S2N–S2P). Moreover, in Sam50 KD cells, mitochondrial cristae were nearly parallel to the IBM (Fig. 4G), and mitochondrial crista dynamic events, including elongation, shortening, and fusion with IBM, were markedly decreased (Fig. 4I and Supplementary Video 9). Additionally, Sam50 KD impairs mitochondrial membrane biogenesis and organization, which may affect MitoTracker Green FM staining and imaging of Hessian-SIM in living Sam50 KD cells (Supplementary Video 9).

By co-IP and mass spectrometry analysis, we identified a new MICOS-interacting protein, ATAD3A (Fig. S2S and supplementary Table 1). ATAD3A KO resulted in abnormal crista morphology and reduced the number of mitochondrial cristae but led to a significant increase in the number of CJs per mitochondrial crista (Fig. 4G and S2N–S4P), indicating that ATAD3A is involved in maintaining CJ. Moreover, ATAD3A KO resulted in increased mitochondrial crista–IBM and crista–crista contact (Fig. 4G–I and and Supplementary Video 10). Occasionally, the mitochondrial cristae network was found in ATAD3A KO HeLa cells (Fig. 4G). These data indicate that ATAD3A negatively regulates the contact and subsequent fusion of mitochondrial cristae with the IBM or adjacent mitochondrial cristae. Therefore, mitochondrial crista–IBM and crista–crista contacts play an important role in regulating mitochondrial crista morphology. Additionally, ATAD3A depletion remarkably reduced the levels of OPA1, Yme1L, and Mic60 (Fig. S2T), suggesting that ATAD3A may cooperate with OPA1, Yme1L and MICOS to regulate mitochondrial crista morphology.

We also used actinomycin D (ActD), a potent inducer of apoptosis, for physiological stimulation to analyze mitochondrial crista morphology. ActD treatment induced rapid mitochondrial fission in HeLa cells, leading to a dramatically increased number of fragmented mitochondria (Figs. S3A–S3B). Moreover, ActD treatment reduced the number of mitochondrial cristae and significantly increased the number of abnormal mitochondrial cristae (Figs. S3C–S3E). Additionally, the number of CJs per crista remained unchanged, but the number of crooked cristae and the cristae width were increased in ActD-treated cells (Figs. S3C, S3F, and S3G). These data suggest that ActD treatment induces changes in mitochondrial crista shape.

### Incomplete mitochondrial fusion contributes to the formation of “cut-through crista” that are maintained by the MICOS complex

**Fig. 5:**
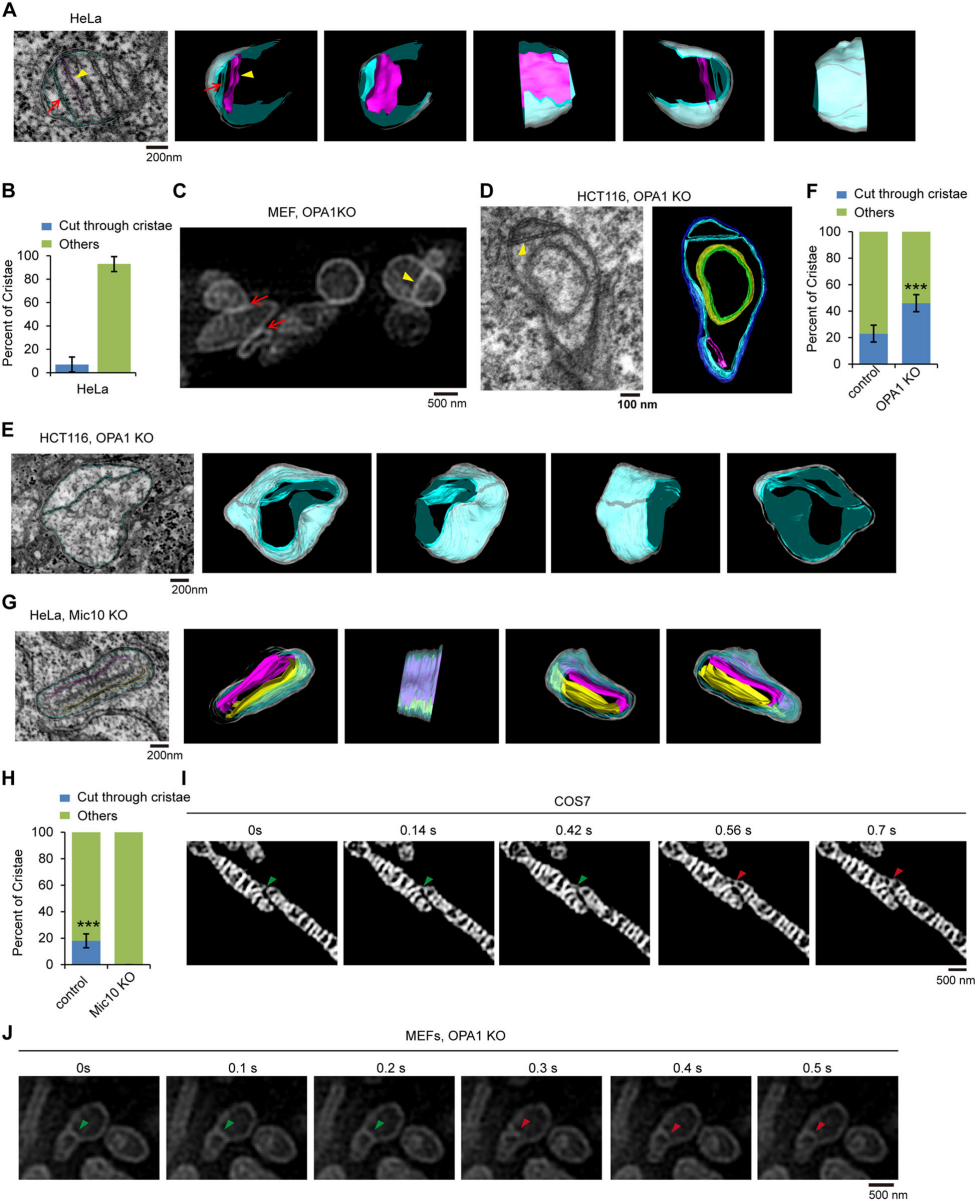
Identification of mitochondrial “cut-through crista”. **A** Focused ion beam/scanning electron microscopy (FIB-SEM) image of mitochondria in HeLa cells. 3D reconstruction and segmentation of mitochondrion showing normal crista and cut-through crista from FIB-SEM were completed by using 3D-IMOD software. White, mitochondrial outer membrane; cyan, mitochondrial IBM and “cut-through crista”; purple red, lamellar crista. **B** Quantification of the “cut-through crista” in mitochondria of HeLa cells (*n* = 100 cristae). Error bars indicate the means ± SD of three independent experiments. **C** Live OPA1 KO MEFs were stained with MitoTrack Green and then imaged by Hessian-SIM. **D** The 70 nm section of specimen of HCT116 OPA1 KO cells was analyzed and imaged by Fei Tecnai Spirit Transmission Microscope. 3D reconstruction and segmentation of mitochondrion were completed using 3D-IMOD software. Blue, mitochondrial outer membrane; cyan, mitochondrial IBM and “cut-through crista”; green, onion-like crista; purple red, tubular crista. **E** Representative thin-section FIB-SEM images of mitochondrion showing cut-through crista with 2 crista junctions in HCT116 OPA1 KO cells. 3D tomographic reconstruction and segmentation of OPA1 KO mitochondrion from FIB-SEM were performed by using 3D-IMOD software. White, mitochondrial outer membrane; cyan, mitochondrial IBM and “cut-through crista”. **F** Quantification of the “cut-through crista” in mitochondria of control and OPA1 KO HCT116 cells (*n* = 100 cristae). “others” means “other types of crista except for cut-through crista”. Statistical significance was assessed from the student’s t-test; error bars indicate the means ± SD of three independent experiments, **p < 0.01 versus control. **G** The continuous 10 nm-thick sections (total 300 nm) from Mic10 KO HeLa cells were analyzed and imaged by FIB-SEM. 3D tomographic reconstruction and segmentation of mitochondrion from FIB-SEM were performed by 3D-IMOD software. White, mitochondrial outer membrane; cyan, mitochondrial IBM; purple red and yellow, lamellar crista without CJ. **H** Quantification of the “cut-through crista” in mitochondria of control and Mic10 KO HeLa cells. “others” means “other types of crista except for cut-through crista” (*n* = 100 cristae). Statistical significance was assessed from the student’s *t*-test; error bars indicate the means ± SD of three independent experiments, ***p < 0.001 versus control. **I** Mitochondria in COS-7 cells were stained with 250 nM MitoTracker Green for 15 min, then were tracked and imaged by time-lapse Hessian-SIM. The green arrowhead indicates the contact and fusion site of two different mitochondria, the red arrowhead indicates the newly formed crista. **J** Live OPA1 KO MEFs were stained with MitoTracker Green (250 nM, 15 min) and imaged by Hessian-SIM. The representative mitochondrial cristae were displayed. The green arrowhead indicates “cut-through crista”, the red arrowhead indicates “lamellar crista”.

Mitochondrial cristae are tethered to the IBM by CJs, which are narrow and ring-like or slot-like structures^3,6^. To further study the morphology of CJs, we used FIB-SEM analysis and 3D tomography to reconstruct the structure of mitochondrial cristae and CJs. In HeLa cells, most mitochondrial cristae are lamellar (Fig. 5A and Supplementary Video 11). Interestingly, HeLa mitochondria contain a few crista-like inner-membrane compartments that fully connect the IBM and open to the inner membrane space (IMS) (Fig. 5A, B and Supplementary Video 11). The distance (~30 nm) between the two membranes of this compartment is very similar to the distance between lamellar cristae (Fig. 5A). Therefore, we named this inner-membrane compartment a cut-through crista. Cut-through crista does not contain a crista tip, and its CJ surround the edge of crista in a long groove. We previously demonstrated that outer and inner mitochondrial membrane fusion are separate processes and that OPA1 mediates inner membrane fusion^16^. According to the morphological characteristics of cut-through crista, we hypothesized that the formation is probably due to the lack of inner mitochondrial membrane fusion after outer mitochondrial membrane fusion. We then analyzed the inner mitochondrial membrane structure in OPA1 KO cells lacking inner mitochondrial membrane fusion. The Hessian-SIM assay revealed that some OPA1 KO mitochondria were closely connected together (the red arrow indicates the tether site) (Fig. 5C), and a cut-through crista (yellow arrowhead indicated) was also formed in OPA1 KO mitochondria (Fig. 5C). Moreover, 3D tomographic reconstruction confirmed the existence of a cut-through crista in the OPA1 KO mitochondrion, and OPA1 KO mitochondria had a larger proportion of cut-through crista than did WT mitochondria (Fig. 5D–F and Supplementary Video 12), suggesting that OPA1 dysfunction promotes the formation of cut-through crista. Overall, outer mitochondrial membrane fusion and the subsequent absence of inner mitochondrial membrane fusion induce the formation of cut-through crista.

We also investigated the role of the MICOS complex in cut-through crista formation by FIB-SEM, and 3D tomographic reconstruction. Mic10, a key component of the MICOS complex, plays a central role in CJ formation (Fig. 4G and S2P). In Mic10 KO mitochondria, cut-through crista were not observed (Fig. 5G–H and Supplementary Video 13), indicating that the MICOS complex is required for the maintenance of cut-through crista.

To directly study the process of cut-through crista formation, inner mitochondrial membrane remodeling during mitochondrial fusion in living cells was tracked by Hessian-SIM. Normally, mitochondrial fusion does not lead to the formation of new mitochondrial cristae in HeLa cells (Fig. S4A). However, sometimes, two mitochondria contact and fuse to form a cut-through crista, probably due to loss of inner mitochondrial membrane fusion (Fig. 5I and S4B). Additionally, approximately 10–15% of mitochondrial fusion leads to cut-through crista in WT cells. Interestingly, by tracking OPA1 KO mitochondria, we observed that certain cut-through crista (just one side) could detach from the IBM and form a lamellar crista (Fig. 5J), suggesting that cut-through crista may be the preform of lamellar cristae.

Together, according to our findings, we propose a mode of cut-through crista formation due to incomplete mitochondrial fusion (Fig. S4C–S4D).

### Spherical crista, a new type of mitochondrial crista, is formed due to incomplete mitochondrial fusion and loss of CJ

**Fig. 6:**
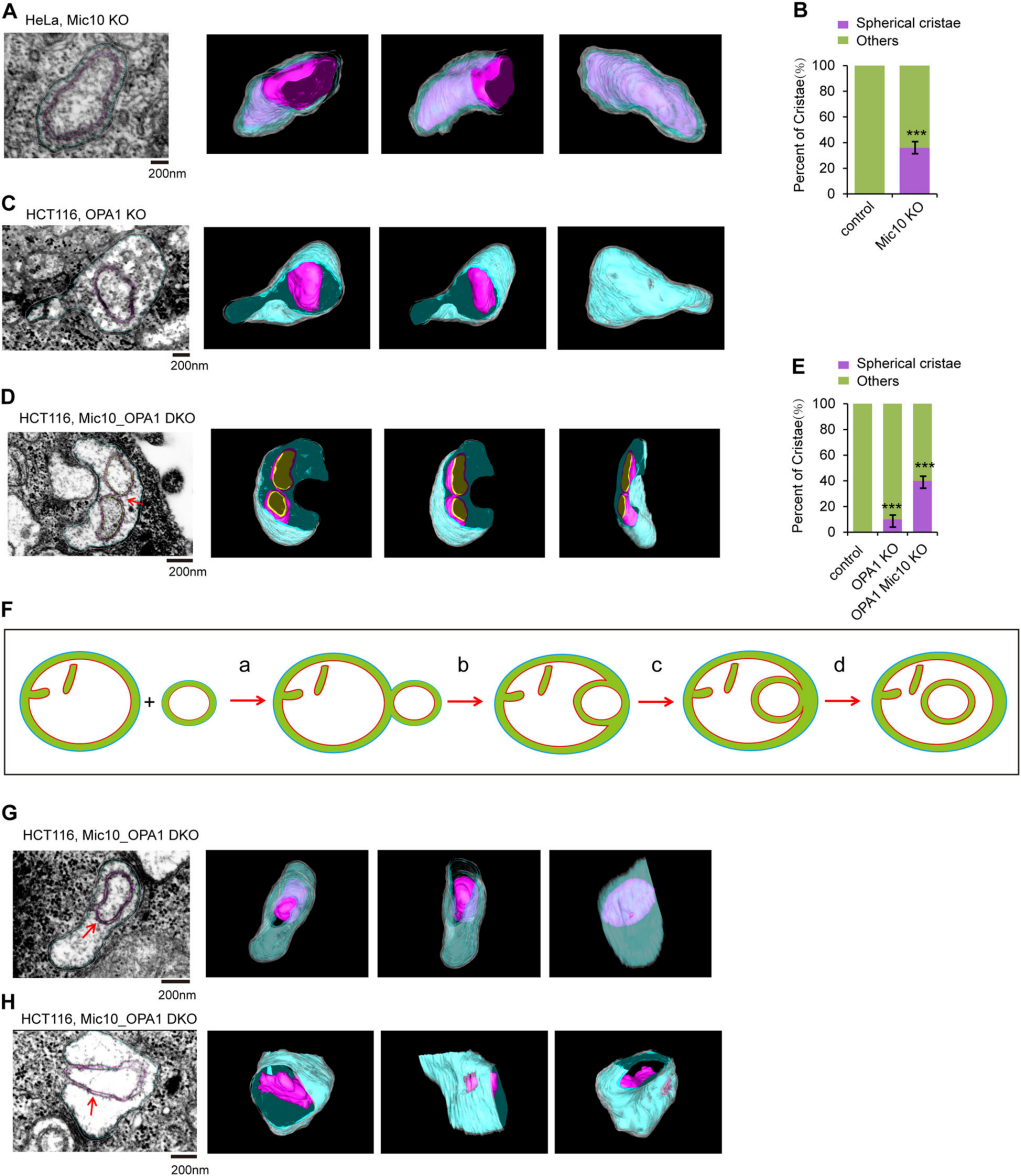
Identification of mitochondrial “spherical crista”. **A** Representative FIB-SEM image of mitochondria in HeLa Mic10 KO cells (left panel). 3D reconstruction and segmentation of mitochondrion showing spherical crista from FIB-SEM were completed by using 3D-IMOD software. White, mitochondrial outer membrane; cyan, mitochondrial IBM; purple red, the spherical crista. B Quantification of the “spherical crista” in mitochondria of control and Mic10 KO HeLa cells. “others” means “other types of crista except for spherical crista” (*n* = 100 cristae). Statistical significance was assessed from the student’s *t*-test; error bars indicate the means ± SD of three independent experiments, ***p < 0.001 versus control. **C** The specimen from HCT116 OPA1 KO cells was analyzed and imaged by FIB-SEM. 3D reconstruction and segmentation of mitochondrion containing spherical crista were performed by using 3D-IMOD software. White, mitochondrial outer membrane; cyan, IBM; purple red, the spherical crista. **D** The samples of HCT116 Mic10-OPA1 DKO cells were analyzed and imaged by FIB-SEM. 3D reconstruction and segmentation of mitochondrion showing two contacted spherical cristae were completed using 3D-IMOD software. White, mitochondrial outer membrane; cyan, mitochondrial IBM; purple red, the spherical crista. **E** Quantification of the spherical crista in mitochondria of control, OPA1 KO, and Mic10-OPA1 double knockout (DKO) HCT116 cells (*n* = 100 cristae). “others” means “other types of crista except for spherical crista”. Statistical significance was assessed from the student’s *t*-test; error bars indicate the means ± SD of three independent experiments, ***p < 0.001 versus control. **F** The mode of the spherical crista formation. Two mitochondria contact and process mitochondrial outer membrane fusion, the small mitochondrion is then engulfed into the bigger mitochondria, and detaches from the IBM of the bigger mitochondrion to form the spherical crista. **G**, **H** Representative FIB-SEM images of mitochondria (**G**, **H**) in HCT116 Mic10-OPA1 DKO cells. 3D reconstruction and segmentation of mitochondrion showing the spherical crista were completed using 3D-IMOD software. White, mitochondrial outer membrane; cyan, mitochondrial IBM; purple red, the spherical crista.

Mic10 is required for CJ formation. The TEM images displayed some onion-like cristae in Mic10 KO mitochondria (Fig. S5A). Surprisingly, FIB-SEM and 3D tomographic reconstruction of the onion-like cristae in the Mic10 KO mitochondrion formed a new type of mitochondrial crista, a “spherical crista”, which is a double-membrane sphere and lacks CJs (Fig. 6A–B and Supplementary Video 14). Only 20–30 percent of the onion-like cristae displayed spherical crista after 3D tomographic reconstruction. Additionally, spherical crista also existed in OPA1 KO mitochondria (Fig. 6C and Supplementary Video 15). These results demonstrate that OPA1 or Mic10 depletion promotes the formation of spherical crista. Then, we investigated the effect of OPA1-Mic10 double knockout (DKO) on mitochondrial crista shape. OPA1-Mic10 DKO mitochondria contain many onion-like cristae (Fig. S5B), and the number of spherical crista was significantly increased in OPA1-Mic10 DKO mitochondria compared with control or OPA1 KO mitochondria (Fig. 6D–E and Supplementary Videos 21–22). Moreover, the spherical crista could contact and fuse with each other in OPA1-Mic10 DKO mitochondria (Fig. 6D and Supplementary Video 16), suggesting that OPA1 is not required for mitochondrial crista membrane fusion. Furthermore, in addition to the spherical crista, a few cut-through cristae also existed in OPA1-Mic10 DKO mitochondria (Figs. S5C–S5D). These findings indicate that cut-through crista are probably preforms of spherical crista. Because spherical crista is detected in Mic10 KO cells, which still display normal tubular mitochondria (Fig. 4H), the formation of spherical crista is not the consequence of mitochondrial fragmentation.

Therefore, we propose the following mechanism of spherical crista formation: after outer membrane fusion between a large and a small mitochondrion, the small spherical membrane coming from the inner membrane of the small mitochondrion is sequestered by the IBM of the large mitochondrion and then detached from the IBM to form a spherical crista, whose membranes arise from two originally distinct inner mitochondrial membranes (Fig. 6F). During this process, the deficiency in inner mitochondrial membrane fusion and impaired MICOS activity mainly contribute to the formation of spherical crista.

**Fig. 7:**
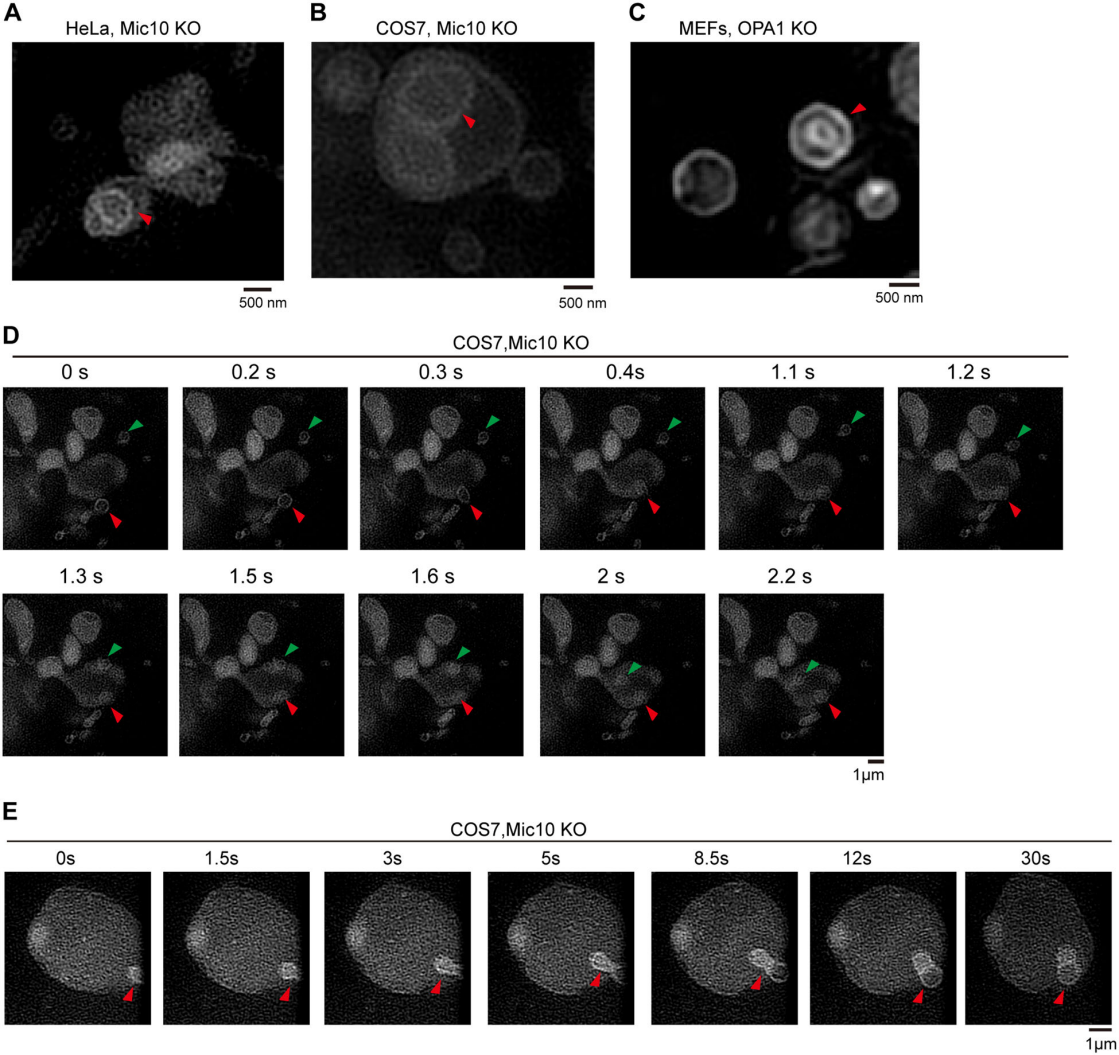
The formation of mitochondrial “spherical crista”. **A**–**C** Live HeLa Mic10 KO (**A**), COS7 Mic10 KO (**B**), and OPA1 KO MEFs (**C**) cells were stained with MitoTracker Green (250 nM, 15 min), and then imaged by Hessian-SIM. The red arrowhead indicates the spherical crista. **D** Representative live-cell time-lapse images of mitochondria in COS7 Mic10 KO cells stained with MitoTracker Green (250 nM, 15 min). Time-lapse Hessian-SIM images reveal that two small mitochondria (the green and red arrowheads indicated) underwent the process of entering into a large mitochondrion. **E** Representative live-cell time-lapse Hessian-SIM images of mitochondria in COS7 Mic10 KO cells stained with MitoTracker Green (250 nM, 15 min). Time-lapse Hessian-SIM images reveal that a small mitochondrion (the red arrowhead indicated) entered into a giant mitochondrion to form a spherical crista.

To verify our hypothesis, we attempted to capture TEM images of key steps (steps “c” and “d”) during the formation of spherical crista. The continuous 10-nm-thick EM sections from the cells were analyzed. We observed that the outer membrane of the onion-like crista is still tethered to the IBM in some continuous sections of the OPA1-Mic10 DKO EM sample (Fig. S6), but before or after these sections, the onion-like crista is completely detached from the IBM (Fig. S6). Further 3D tomographic reconstruction showed that the outer membrane of the spherical crista partly connects to the IBM (Fig. 6G, H, and Supplementary Videos 17–19), suggesting that this spherical crista is in its final stages of formation and has not yet completely detached from the IBM. Additionally, Hessian-SIM imaging showed that the spherical crista was observed in living OPA1 KO and Mic10 KO cells (Fig. 7A–C). We then used time-lapse Hessian-SIM to track the process of spherical crista formation in living cells. Some small spherical mitochondria fused with and entered the large spherical mitochondrion to form spherical crista in Mic10 KO cells (Fig. 7D, E and Supplementary Videos 20–21). These findings strongly confirm the mode of spherical crista formation (Fig. 6F).

Although few mitochondrial spherical cristae are observed in normal HeLa and HCT116 cells, onion-like mitochondrial cristae (2D form of spherical crista) are frequently observed in the mitochondria of cells with mtDNA mutations, deletion or loss^38,39^. We then investigated mitochondrial crista morphology in 143B Rho^0^ (ρ^0^) cells lacking mtDNA. 143B Rho^0^ mitochondria displayed remarkably increased onion-like mitochondrial cristae (Figs. S7A–S7C), indicating that mtDNA loss might be involved in the formation of spherical crista.

In addition, OPA1 KO led to a dramatic reduction in MICOS components (Mic60, Mic10, Mic13, and Mic19) and mitochondrial respiratory complex components (SDHA, COX2, and COX4) (Fig. S8A). However, Mic10 KO did not affect MICOS components (Mic60, Mic10, and Mic19) or mitochondrial respiratory complex components (SDHA, COX2, and COX4) (Fig. S8B), indicating that the spherical crista in Mic10 KO mitochondria still contain normal crista proteins, including respiratory complexes and MICOS components. Moreover, OPA1 KO but not Mic10 KO exhibited a decreased mitochondrial membrane potential (Figs. S8C–S8D), suggesting that spherical crista in Mic10 KO mitochondria maintain a normal mitochondrial membrane potential.

## Discussion

Mitochondrial cristae are the main sites of electron transfer and oxidative phosphorylation in mitochondria. The study of mitochondrial crista dynamics and remodeling has been a focus and a challenge in the field of mitochondrial research. The traditional TEM technique can only display the crista morphology of a certain section of a mitochondrial sample and cannot reflect the dynamic changes of mitochondrial cristae in living cells. Our newly established Hessian-SIM technique can clearly present the mitochondrial crista morphology in living cells and track the crista’s dynamic change and formation process by time lapse (Fig. 1 and Supplementary Video 1). However, due to the limitation of its optic resolution, Hessian-SIM cannot distinguish the fusion or contact between mitochondrial cristae and that between cristae and the IBM. Therefore, we used Hessian-SIM combined with TEM, FIB-SEM and 3D tomographic reconstruction to study mitochondrial crista dynamics. These technologies complement each other functionally: Hessian-SIM enables visualization of the mitochondrial crista dynamic process in living cells, TEM has the advantage of optical resolution in visualizing the mitochondrial crista ultrastructure but can only capture fixed samples, and FIB-SEM and 3D tomographic reconstruction enables the stereoscopic presentation of mitochondrial cristae. Therefore, the combination of these three techniques is the best choice for studying mitochondrial crista dynamics and will lead to a re-examination of previous concepts about mitochondrial membrane organization and biogenesis.

We discovered two novel types of mitochondrial cristae, cut-through crista and spherical crista (Figs. 3–5). Furthermore, we demonstrated the pathways of cut-through crista and spherical crista formation and proposed the modes of formation of these two types of mitochondrial cristae (Fig. 6F and S4D). OPA1 dysfunction-mediated inhibition of inner mitochondrial membrane fusion contributes to the formation of these 2 types of cristae. Spherical crista mainly existed in OPA1- or MICOS-depleted mitochondria and largely formed in OPA1-Mic10 DKO mitochondria (Fig. 6A–E), but no spherical crista were found in normal mitochondria (Figs. 6B, E), suggesting that spherical crista are highly associated with mitochondrial dysfunction. Spherical crista appear as onion-like structures in 2D EM imaging (Figs. S5A and S5B). Onion-like cristae were largely formed in response to inhibition of oligomerization of F1FO ATP synthase^40^, but oligomerization of F1FO ATP synthase is required for the formation of tubular cristae^6^, indicating that the model of spherical crista formation is different from that of tubular cristae formation. Indeed, the model of spherical crista formation is totally different from the “invagination model” and the “balloon model” (Fig. 6F). Our findings reveal the diversity of mitochondrial crista morphology and multiple pathways for crista formation.

Mitochondrial crista remodeling is highly associated with mitochondrial functions. Mitochondrial crista shape regulates the organization and function of the oxidative phosphorylation system, directly affecting cellular energy metabolism^3,4^. In addition, mitochondrial crista remodeling regulates cytochrome c release during apoptosis^10,13^, and we also observed that mitochondrial crista shape is remodeled in ActD-treated cells (Fig. S3C–S3D). Moreover, OPA1-dependent mitochondrial crista remodeling is required for cellular adaptation to metabolic demand and is critical for cell survival and growth^9^.

## Materials and methods

### Cell culture and reagents

HeLa, HCT116, MEFs, and COS7 cells were cultured in high-glucose DMEM supplemented with 10% FBS (PAN), 100 U/mL penicillin (Gibco) and 100 mg/mL streptomycin (Gibco). Cells were maintained in 5% CO_2_/ 95% air at 37 °C. For Hypoxia exposure, cells were placed in a modulator chamber with 1% O_2_ /5% CO_2_ /94% N_2_ at 37 °C. Lipofectamine 2000 (Invitrogen) were carried out as protocol present. MitoTracker Green and MitoTrakcer Red used for Hessian-SIM imaging were obtained from Invitrogen.

### Hessian structured illumination microscopy (Hessian-SIM) imaging

Hessian-SIM analysis was performed as described previously^27^. Cells used in this study were seeded onto poly-L-lysine-coated coverslips, and cultured for an additional 12–18 h before the SIM imaging. To label the mitochondria, cells were incubated with 250 nM MitoTracker Green FM (Thermo Fisher Scientific) in high-glucose DMEM at 37 °C for 15 min, and then washed with HBSS solution containing Ca^2+^, Mg^2+^ but no phenol red (Thermo Fisher Scientific) three times. Super-resolution images acquired on a Hessian-SIM microscope equipped with a TIRF objective (ApoN 100X/1.7 HI Oil, Olympus) and a multiband dichroic mirror (DM, ZT405/488/561/640-phase R; Chroma), and three lasers (Sapphire488LP-200, Coherent; Sapphire 561LP-200, Coherent; and MRL-III -640-150, IL photonics) as the light sources, and an acoustic optical tunable filter (AOTF, AA Opto-Electronic, France) to combine, switch, and adjust the illumination power of the lasers. Nine raw frames illuminated with a periodic pattern of parallel lines and shifted through three phases for each of three orientation angles were used to reconstruct one SR image. The exposure time was set to 10 ms for each raw images capture. All SIM images were analyzed with the Hessian-SIM microscopy method and the ImageJ software. Four ImageJ plugins were used to improve the SIM images quality.

### Mitochondrial cristae morphology and dynamics analysis

Cells were stained with MitoTracker Green FM (Thermo Fisher Scientific) to label mitochondrial cristae and inner boundary membrane (IBM), and different mitochondrial cristae dynamic events were quantified manually by observing the corresponding Live-Hessian-SIM movies for a time span of 0–10 s at a frame rate of 0.1 s/frame. The number of “Shortening” and “Elongation” events are calculated by the cristae length, each crista that is shortened or elongated beyond 50 nm is considered a dynamic event. The number of “Crista–IBM contact” (mitochondrial crista contact with the inner boundary membrane) and “Crista–Crista contact” (mitochondrial crista contact with another crista) events were observed by continuously cristae change, and the “Crista–IBM contact” or “Crista–Crista contact” events retaining over 2 s (excluding false events due to imaging angle) were recorded and counted. The intact mitochondrial crista containing one or two crista junctions is separated from the inner boundary membrane to form a new crista losing one crista junction, this event is identified as “Cristae detaching from the IBM”. The event in two separate mitochondrial cristae contact and merge into a single crista that retained for more than two seconds (excluding false events due to imaging angle), is called “cristae fusion”. If one mitochondrial crista is divided into two cristae retaining more than two seconds (excluding false events due to imaging angle), it is called “cristae division”. Due to the limitation of SIM resolution, only the contact, fusion, division, or detachment events retained for more than 2 s (excluding false events due to imaging angle) were counted. All quantifications were analyzed by three independent experiments (at least 100 mitochondrial cristae in 10–30 mitochondria from 10–20 cells were analyzed for each experiment or each group). The difference between groups was compared by the student’s *t*-test, the asterisks *, **, and *** denote statistical significance with *P* < 0.05, 0.01, and 0.001, respectively.

### Transmission electron microscopy (TEM) analysis

The procedure of TEM was performed according to the previous report^41^. HeLa, HCT116, and 143B cells were fixed with 3% PFA, 0.5% glutaraldehyde, and 0.25% sucrose in 0.1 M sodium phosphate buffer (PH 7.4) for 1 h at RT, then were washed with 0.1 M PB contain 0.25% sucrose (PH 7.4). Next, cells were collected using a cell scraper and subsequently were gradient centrifuged, the samples were then postfixed with 2% osmium tetroxide in 0.1 M PB at 4 °C for 1 h, 2% uranyl acetate overnight 4 °C. Fixed samples were dehydrated with different concentrations of ethanol, embedded in Questol-812, and polymerized at 60 °C for 48–72 h. The blocks were ultrathin-sectioned at 70 nm with a diamond knife using an ultramicrotome (Leica). Sections were placed on copper grids and stained with 2% Lead citrate at RT for 15 min and then rinsed with the distilled water. The grids were observed and imaged under a JEM-1400Plus (JEOL) transmission electron microscope at an acceleration voltage of 80 kV.

### Focused ion beam/scanning electron microscopy (FIB-SEM) analysis

HeLa and HCT116 cells were fixed, filtrated, and polymerized as described previously^28^. Focused ion beam milling and scanning electron microscopy imaging were carried out with an FEI Helios NanoLab G3 UC (from Tsinghua, China). FIB milling was performed with 600 pA to 20 nA for the given samples. SEM‐Imaging current was 0.4 nA. FIB milling steps was 10 nm/slice, and each slice was imaged. Accordingly, each image represents 10 nm of the stack, at ×35,000 magnification. The selected mitochondria were there-dimensional (3D) reconstituted and 3D rendered by IMOD software.

### SDS-PAGE and western blotting analysis

Cells were harvested by 0.25% trypsin (Gbico), cell suspension was then centrifuged, pellet cells were resuspended with 1× sample buffer, and boiled for 12 min. The protein samples were separated by SDS-PAGE and transferred into 0.45 μm PVDF membranes, 2 h later, membranes were blocked by 5% non-fat milk for 1 h, then incubated with primary antibody 30 min-2h in 5% non-fat milk followed by incubation with 1:5000 HRP secondary antibodies for 1 h. Detection of immunoreactive protein on Fuji x-ray film using ECL chemiluminescent substrate (Bio-Red). The following primary antibodies were used: anti-GAPDH and anti-Beta-Tubulin were obtained from Santa Cruz Biotechnology; anti-COX4, anti-COX2, anti-ATAD3A, anti-Mic60, anti-Yme1L, and anti-Tom20 were from Proteintech; anti-OPA1 was obtained from BD Biosciences, anti-Sam50 was obtained from Abcam; anti-Mic19 was from Abclonal; anti-Mic10 was from Invitrogen.

### Statistical analysis

For statistical analysis, all cells and mitochondria were randomly selected. All statistical data were presented as the mean ± SD in three independent experiments. Student’s *t*-test was used to calculate *P*-values. Statistical significance is displayed as *p < 0.05, **p < 0.01, ***p < 0.001.

## Supplementary information

Figure S1

Figure S2

Figure S3

Figure S4

Figure S5

Figure S6

Figure S7

Figure S8

Supplementary Figure Legends

Supplementary Information

Supplementary Video 01

Supplementary Video 02

Supplementary Video 03

Supplementary Video 04

Supplementary Video 05

Supplementary Video 06

Supplementary Video 07

Supplementary Video 08

Supplementary Video 09

Supplementary Video 10

Supplementary Video 11

Supplementary Video 12

Supplementary Video 13

Supplementary Video 14

Supplementary Video 15

Supplementary Video 16

Supplementary Video 17

Supplementary Video 18

Supplementary Video 19

Supplementary Video 20

Supplementary Video 21
