# Supplementary figures and images for: OPA1 and MICOS Regulate mitochondrial crista dynamics and formation
Source: Cell Death Dis. 2020 Oct 31;11(10):940. doi: 10.1038/s41419-020-03152-y (PMC7603527; doi:10.1038/s41419-020-03152-y)

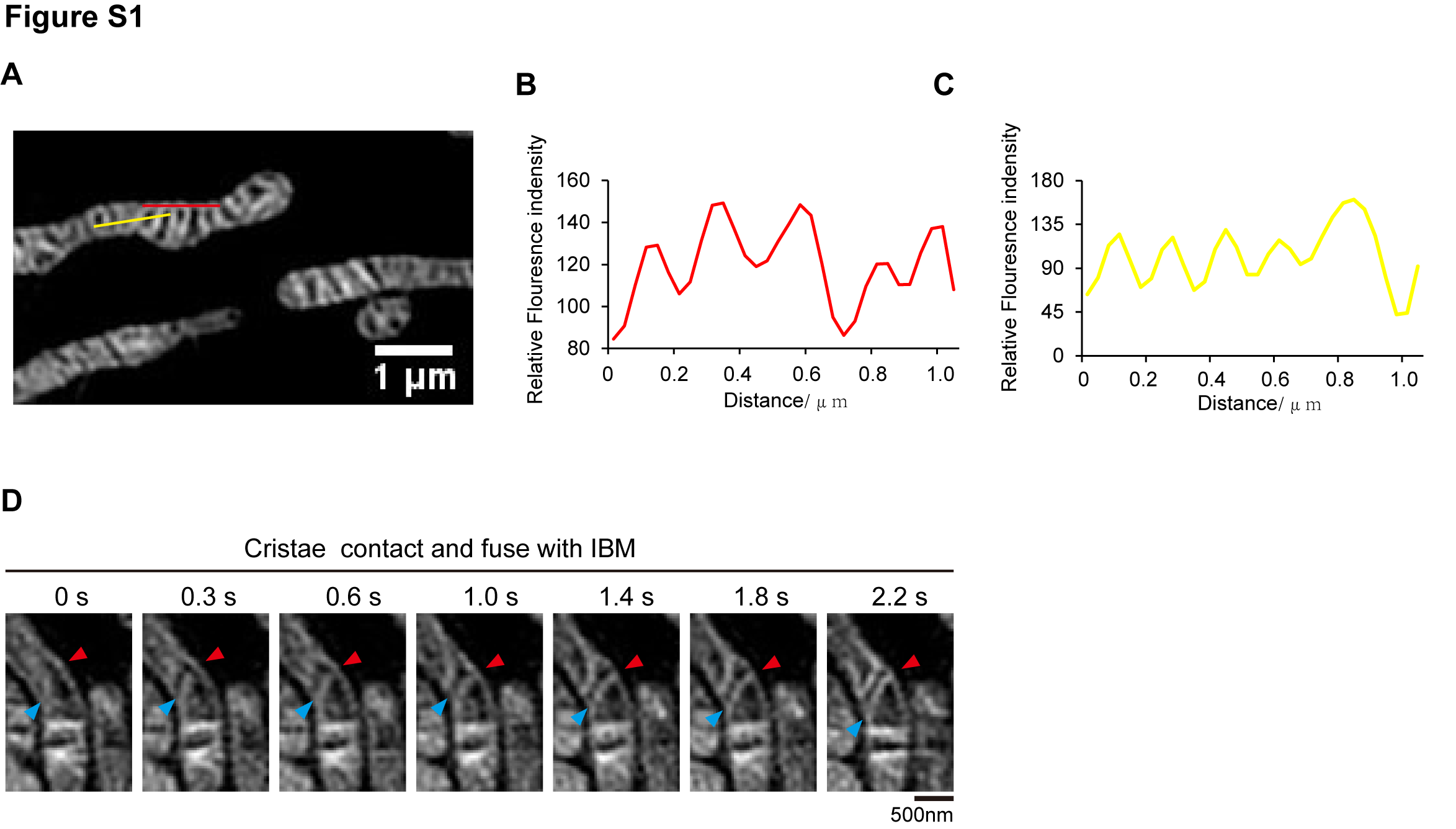

Supplement: Supplementary file 1 — Figure S1 [file 41419_2020_3152_MOESM1_ESM.tif]

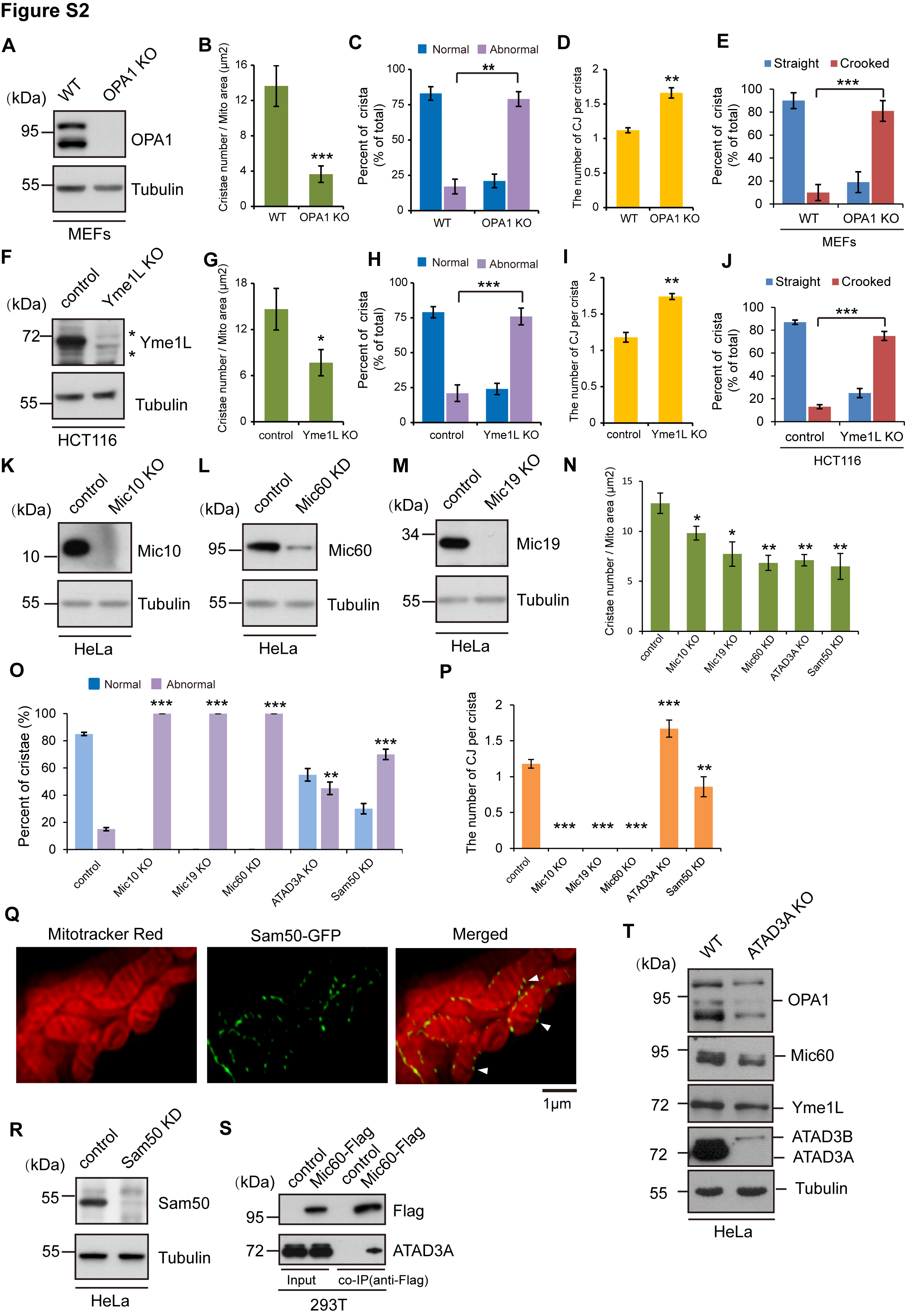

Supplement: Supplementary file 2 — Figure S2 [file 41419_2020_3152_MOESM2_ESM.tif]

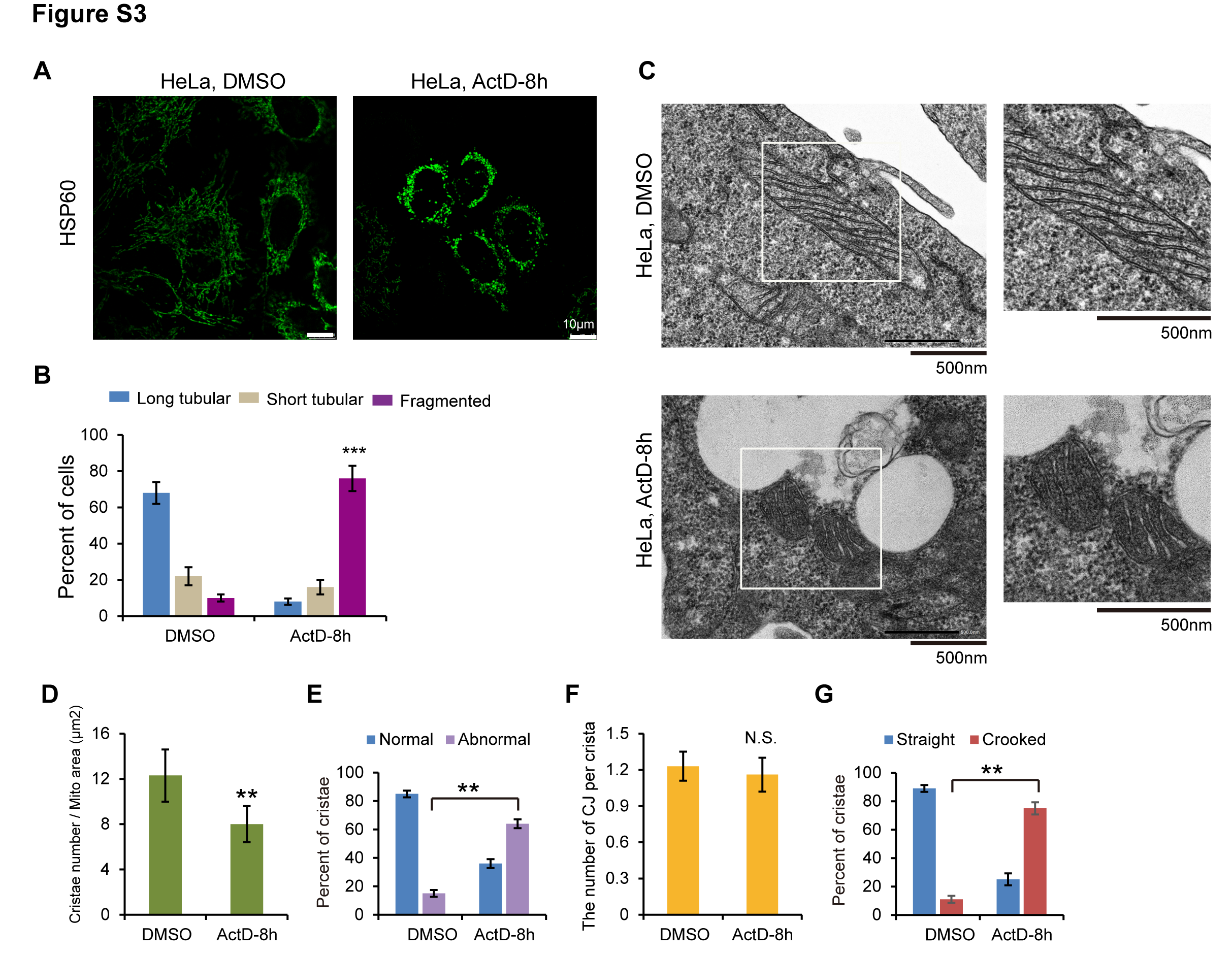

Supplement: Supplementary file 3 — Figure S3 [file 41419_2020_3152_MOESM3_ESM.tif]

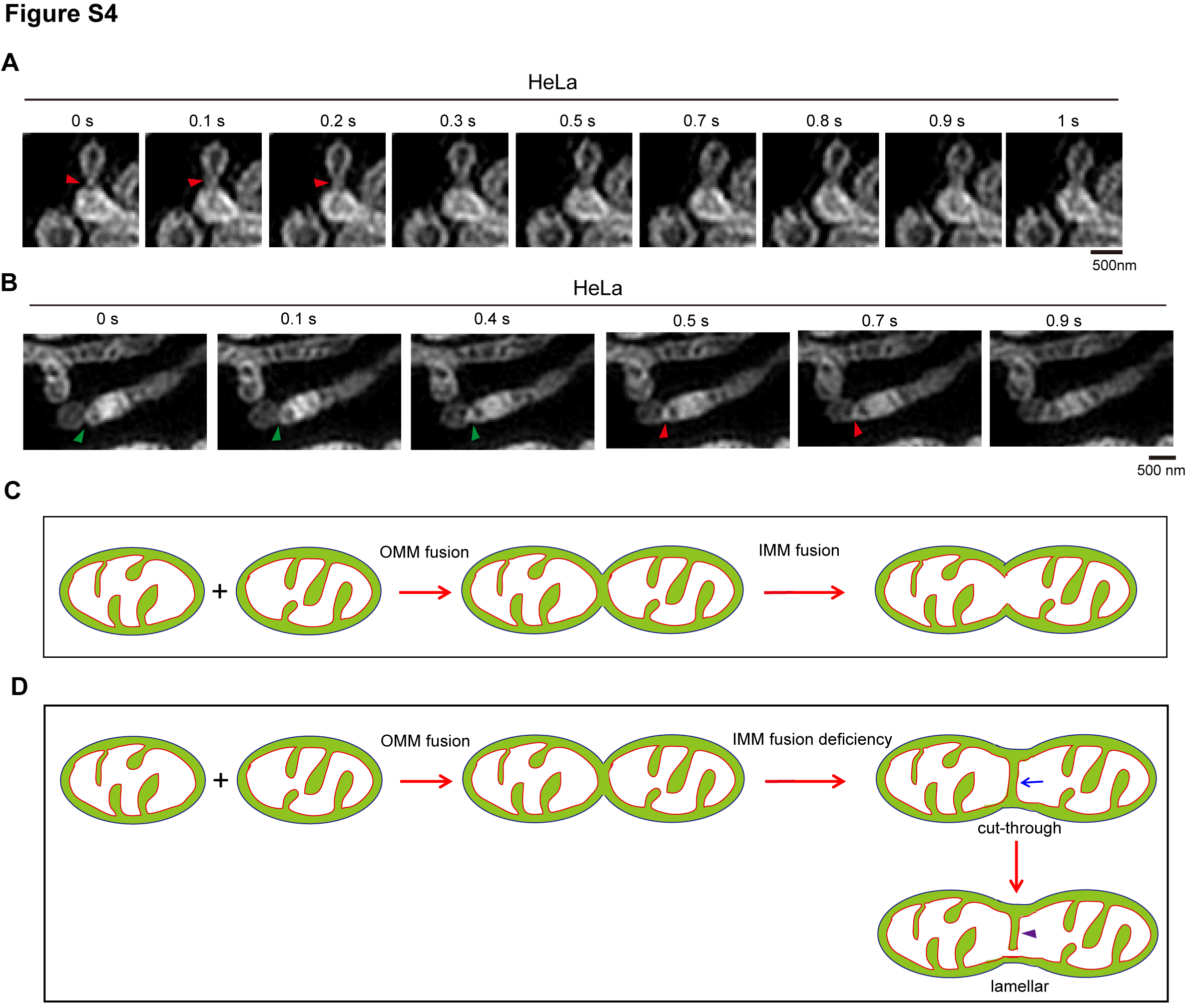

Supplement: Supplementary file 4 — Figure S4 [file 41419_2020_3152_MOESM4_ESM.tif]

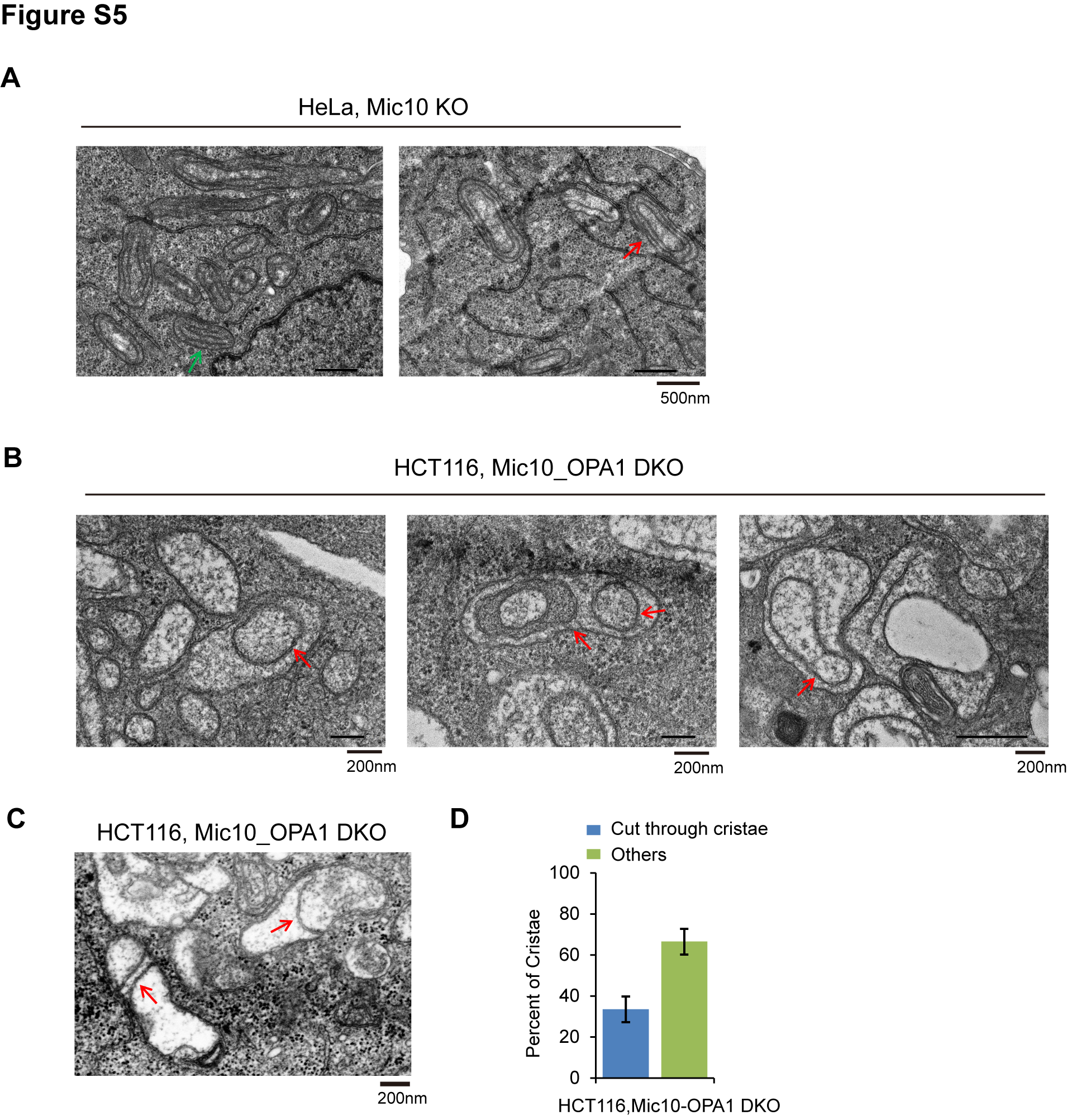

Supplement: Supplementary file 5 — Figure S5 [file 41419_2020_3152_MOESM5_ESM.tif]

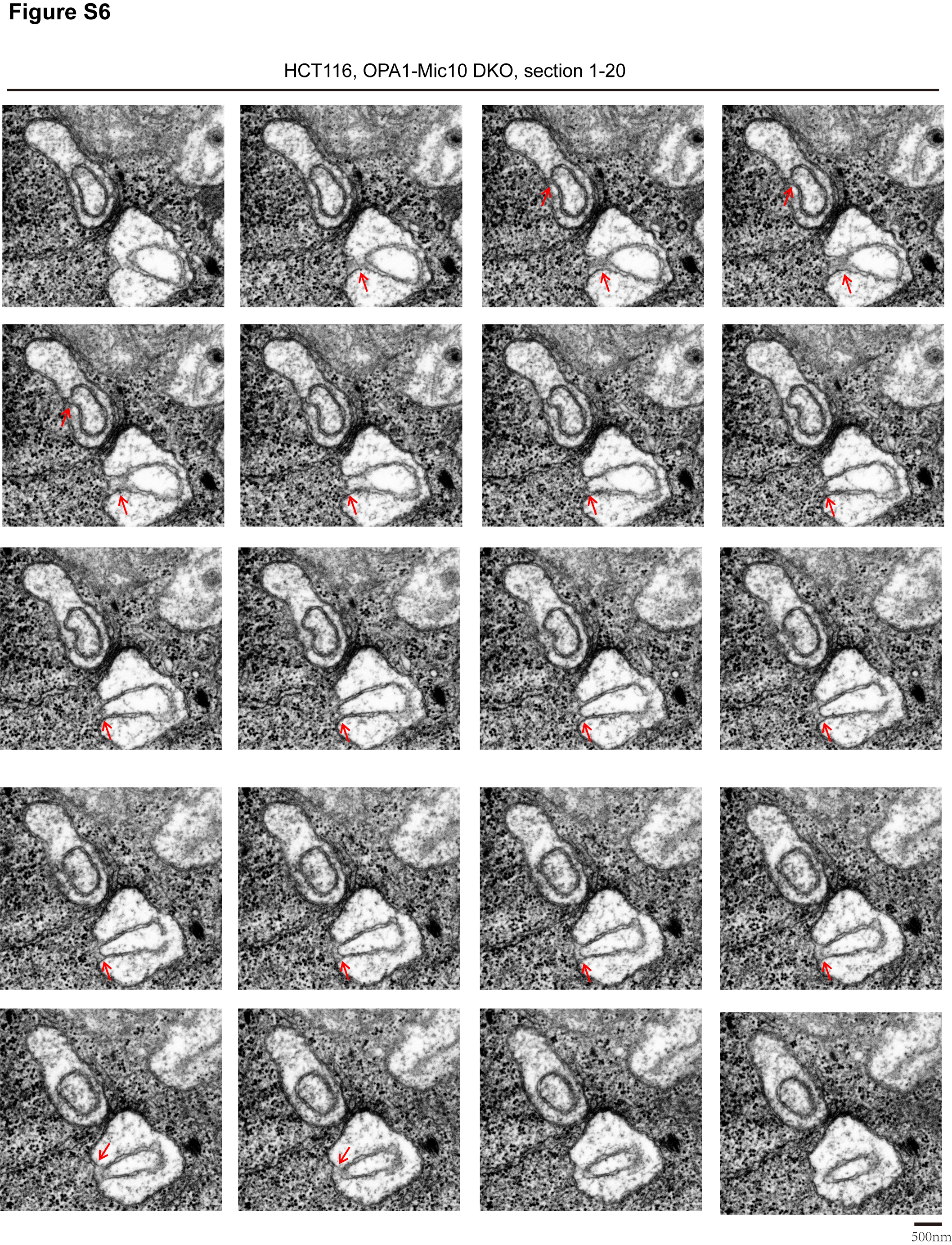

Supplement: Supplementary file 6 — Figure S6 [file 41419_2020_3152_MOESM6_ESM.tif]

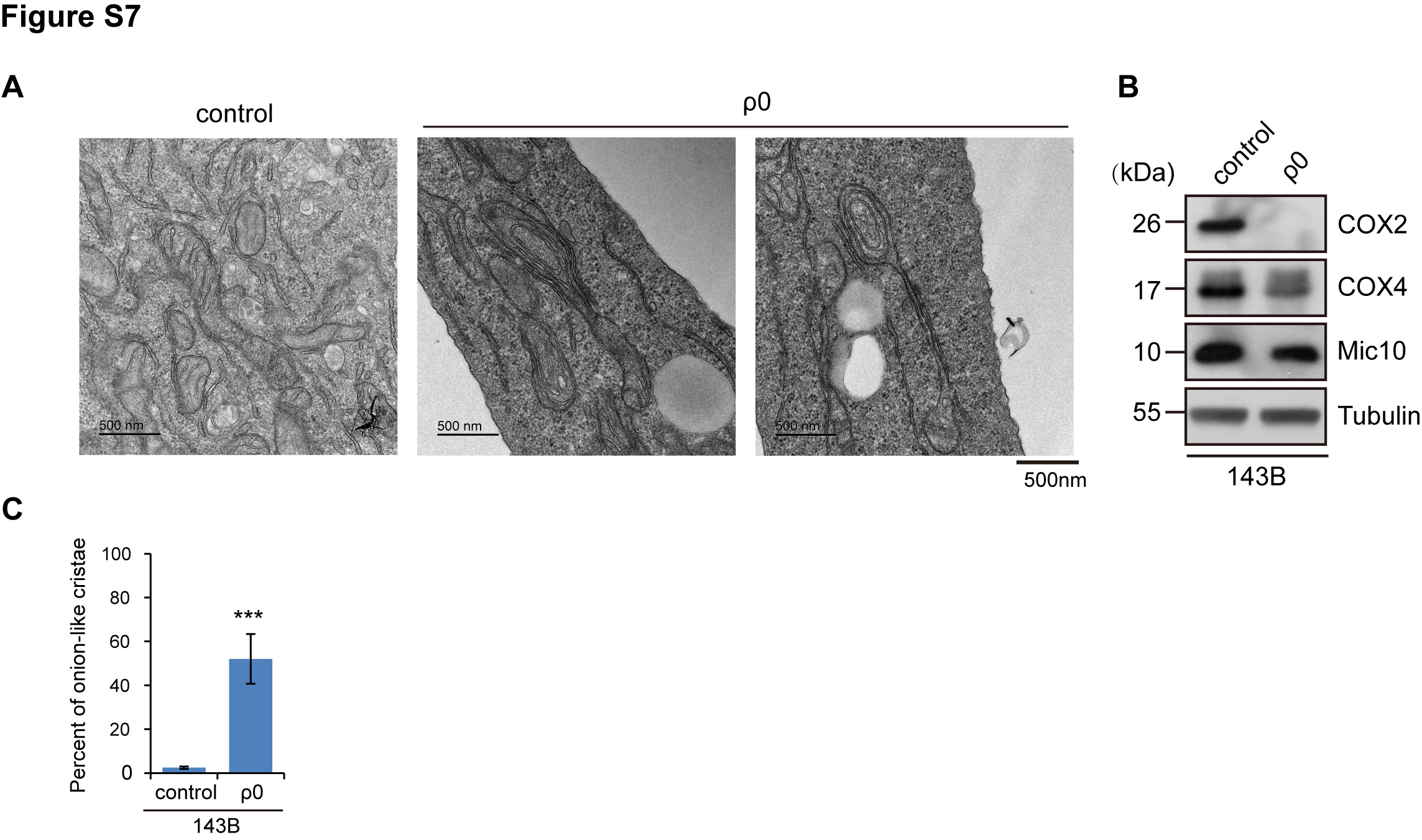

Supplement: Supplementary file 7 — Figure S7 [file 41419_2020_3152_MOESM7_ESM.tif]

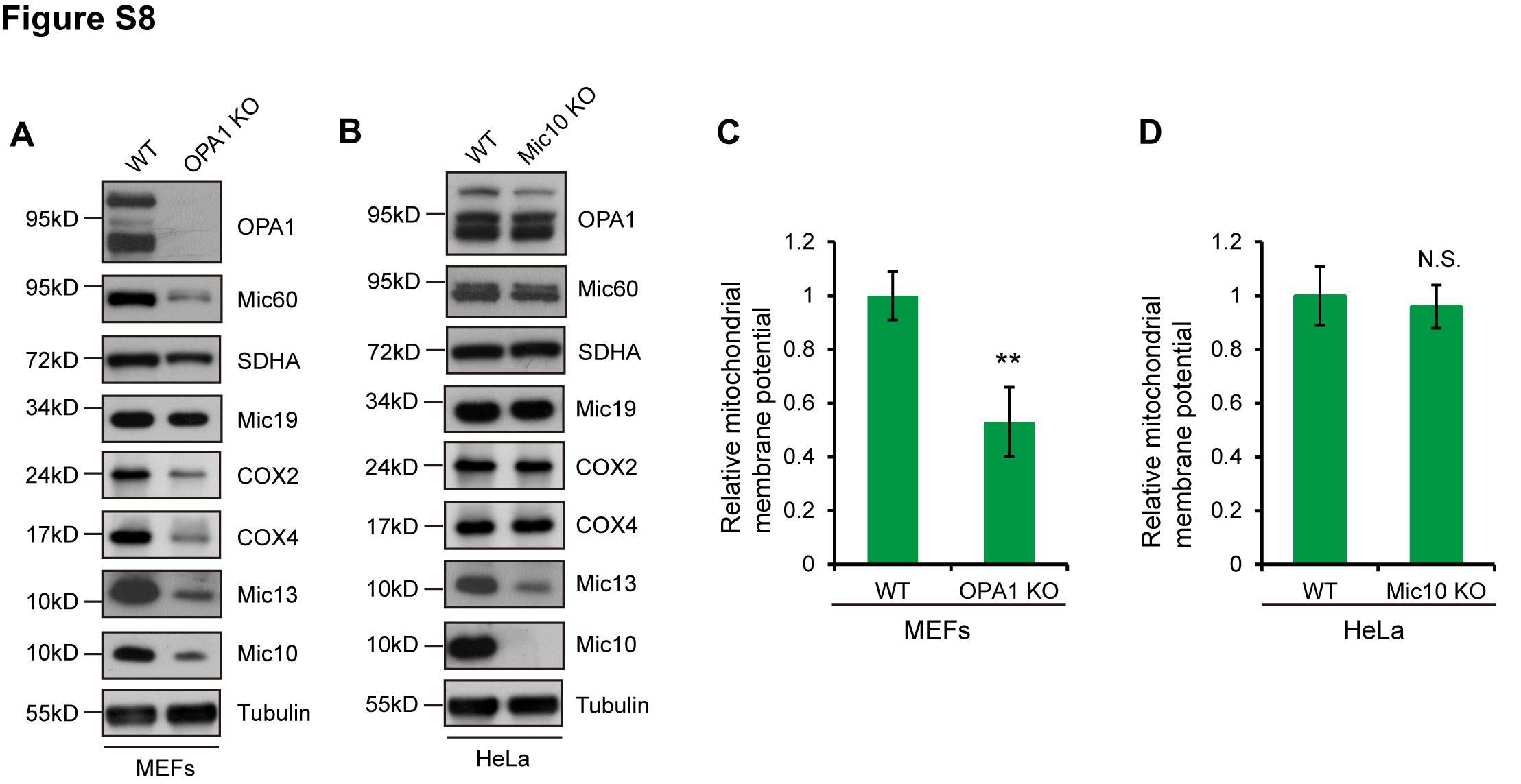

Supplement: Supplementary file 8 — Figure S8 [file 41419_2020_3152_MOESM8_ESM.tif]
