# Supplementary material for: OPA1 and MICOS Regulate mitochondrial crista dynamics and formation
Source: Cell Death Dis. 2020 Oct 31;11(10):940. doi: 10.1038/s41419-020-03152-y (PMC7603527; doi:10.1038/s41419-020-03152-y)
Supplement: Supplementary file 9 — Supplementary Figure Legends [file 41419_2020_3152_MOESM9_ESM.docx]

**SUPPLEMENTARY FIGURE LEGENDS**

**Figure S1. The fluorescence intensity of mitochondrial IBM and cristae stained with MitoTracker Green**

(A) Representative live-cell time-lapse Hessian-SIM images of mitochondrial in HeLa cells stained with MitoTracker Green (250nM, 15min). Red line indicates mitochondrial IBM; yellow line indicates mitochondrial crista.

(B-C) The pixel intensity of the red (B) and yellow (C) lines in “A”.

(D) Representative live-cell time-lapse images of mitochondrial cristae contacting and fusing with IBM. HeLa cells were treated with MitoTracker Green (250nM, 15min) and tracked by time-lapse Hessian-SIM. The red arrowheads showed that the site of mitochondrial cristae contacting and fusing with the IBM.

**Figure S2. The regulators of mitochondrial crista dynamics**

(A) Cell lysates of WT MEFs, OPA1 KO MEFs were analyzed by Western blotting using the antibodies against OPA1 or Tubulin.

(B-E) Mitochondrial ultrastructure of WT MEFs and OPA1 KO MEFs were analyzed by TEM. The number of mitochondrial cristae (B), abnormal cristae (C), the number of CJ per crista (D), and mitochondrial cristae morphology (E) were quantified (three independent experiments, 100 mitochondria cristae for each experiment). Statistical significance was assessed from the student’s t-test; error bars represent means ± SD of three independent experiments, **p< 0.01, ***p< 0.001 versus control.

(F) Cell lysates of control and Yme1L KO HCT116 cells were analyzed by Western blotting using the indicated antibodies.

(G-J) Mitochondrial ultrastructure of control and Yme1L KO HCT116 cells were analyzed by TEM. The number of mitochondrial cristae (G), abnormal cristae (H), the number of CJ per crista (I), and mitochondrial cristae morphology (J) were quantified (three independent experiments, 100 mitochondria cristae for each experiment). Statistical significance was assessed from the student’s t-test; error bars represent means ± SD of three independent experiments, **p< 0.01,***p< 0.001 versus control.

**(K-M)** Cell lysates of control, Mic10 KO (K), Mic60 KD (L), Mic19 KO (M) HeLa cells were analyzed by Western blotting using the indicated antibodies.

(N-P) Mitochondrial ultrastructure of 6 indicated cells was analyzed by TEM. The number of mitochondrial cristae (N), abnormal cristae (O), and the number of CJ per crista (P) were quantified (three independent experiments, 100 mitochondria cristae for each experiment). Statistical significance was assessed from the student’s t-test; error bars indicate the means ± SD of three independent experiments, * p< 0.05, **p< 0.01,***p< 0.001 versus control.

(Q) Representative live-cell time-lapse Hessian-SIM images of mitochondria in Sam50-GFP expressed HeLa cells stained with Mito Tracker Green (250nM, 15min). Time-lapse Hessian-SIM images reveal that Sam50-GFP displays a puncta localization facing mitochondrial crista.

**(R)** Cell lysates of control and Sam50 KD HeLa cells were analyzed by Western blotting using anti-Sam50 or anti-Tubulin antibodies.

(S) Cell lysates of 293T cells expressing Mic60-Flag were used to perform co-immunoprecipitation (co-IP) assay using anti-Flag **M2 resin. The final eluted products were analyzed by Western blotting using anti-Flag, or anti-ATAD3A antibodies.**

**(T)** Cell lysates of control and ATAD3A KO HeLa cells were analyzed by Western blotting using the indicated antibodies.

**Figure S3. HeLa cells treated with apoptosis inducer-Actinomycin D**

(A) HeLa cells were treated with or without Actinomycin D for 8h, and then were immunostained for HSP60 (green, anti-HSP60). Cells were visualized and imaged by confocal microscopy. Boxed regions in the left image are magnified.

(B) Mitochondrial morphology described in “A” was counted according to the criteria detailed. All data represent the means ± SD of three independent experiments (100 cells per independent experiment). Statistical significance was assessed by the student’s t-test, ***P < 0.001 and N.S indicates none significance.

(C) Representative TEM images of mitochondria in control and HeLa cells treated with 4mM Actinomycin D for 8h.

(D-G) Mitochondrial ultrastructure of HeLa WT and HeLa cells treated with ActD 8h were analyzed by TEM. The number of mitochondrial cristae (D), abnormal cristae (E), the number of CJ per crista (F), and mitochondrial cristae morphology (G) were quantified. Statistical significance was assessed from the student’s t-test; error bars represent means ± SD of three independent experiments, **p< 0.01, ***p< 0.001 versus control.

**Figure S4. Formation of “cut-through crista”.**

(A) Representative live-cell time-lapse Hessian-SIM images of mitochondria in HeLa cells. Mitochondria in HeLa cells were stained with MitoTracker Green (250nM, 15min), then were tracked and imaged by time-lapse Hessian-SIM. The indicated images demonstrate completely mitochondrial fusion between two mitochondria. the red arrowhead indicates the fusion site.

(B) Mitochondria in HeLa cells were stained with 250nM MitoTracker Green for 15min, then were tracked and imaged by time-lapse Hessian-SIM. The green arrowhead indicates the contact and fusion site of two different mitochondria, the red arrowhead indicates the newly formed crista.

(C-D) The mode of “cut-through crista” formation. Two mitochondria contact and firstly process mitochondrial outer membrane fusion, then undergo inner membrane fusion (C); however, if mitochondrial inner membrane fusion is inhibited, “cut-through crista” is formed (D). In addition, “cut-through crista” could change to the lamellar crista due to detachment from IBM.

**Figure S5. The role of Mic10 and OPA1 in mitochondrial cristae remodeling**

(A) Representative TEM images of mitochondrial ultrastructure in HeLa Mic10 KO cells. The green arrow indicates mitochondrial crista lacking Cj, the red arrow indicates mitochondrial onion-like crista.

(B) Representative TEM images of mitochondrial ultrastructure in HCT116 Mic10_OPA1 DKO cells. The red arrow indicates a mitochondrial onion-like crista.

(C) Representative TEM images of mitochondrial ultrastructure in HCT116 Mic10_OPA1 DKO cells. The red arrow indicates a mitochondrial cut-through crista.

(D) Quantification of mitochondrial cut-through crista in mitochondria (n=30) of HeLa Mic10_OPA1 DKO cells (n=30 mitochondrial crista). “others” means “other types of crista except for cut-through crista”.

**Figure S6. Mitochondrial spherical crista formation**

Continuous FIB-SEM images of the thin-section specimen (10nm/section) of HCT116 Mic10-OPA1 DKO cells. FIB-SEM images reveal a certain phase during mitochondrial spherical cristae formation. The red arrow indicates the connection between mitochondrial spherical cristae and IBM.

**Figure S7. Mitochondrial cristae morphology in ρ^0^ 143B cells**

(A) Representative TEM images of mitochondria in control or ρ^0^ 143B cells (mtDNAs are absent in ρ^0^ cells).

(B) Cell lysates of control orρ^0^ 143B cells were analyzed by Western blotting using antibodies against COX2, COX4, Mic10, and Tubulin.

(C) Quantification of the “onion-like crista” in mitochondria (n=100) of control or ρ^0^ 143B cells. Statistical significance was assessed from the student’s t-test; error bars indicate the means ± SD of three independent experiments, ***p< 0.001 versus control.

**Figure S8. The effect of Mic10 KO or OPA1 KO on other related proteins and mitochondrial membrane potential**

(A) Western blotting analysis of MEFs WT or OPA1 KO cells lysates using the indicated antibodies.

(B) Western blotting analysis of HeLa control or Mic10 KO cells lysates using the indicated antibodies.

(C-D) WT and OPA1 KO MEFs (C), or control and Mic10 KO cells (D) were incubated with a mitochondrial membrane potential-dependent dye, TMRM (tetramethylrhodamine methyl ester, 250 nM, 30 min), mitochondrial membrane potential was then analyzed and quantified by FACS, and the related mitochondrial membrane potential was displayed.
