# Supplementary material for: OPA1 and MICOS Regulate mitochondrial crista dynamics and formation
Source: Cell Death Dis. 2020 Oct 31;11(10):940. doi: 10.1038/s41419-020-03152-y (PMC7603527; doi:10.1038/s41419-020-03152-y)
Supplement: Supplementary file 10 — Supplementary Information [file 41419_2020_3152_MOESM10_ESM.docx]

**SUPPLEMENTARY INFORMATION**

**MATERIALS AND METHODS**

**Plasmids and RNA interference**

Homo Mic60 cDNA was cloned into a modified pMSCV-puro (Addgene) constructs containing a C-terminal 3× FLAG tag. In brief, the shRNA target sequences against human Sam50 or Mic60 were subcloned into a modified retroviral vector with the H1 promoter to drive the expression of shRNAs ^[1](#_ENREF_1)^. HeLa cells infected with retrovirus were selected by 1 µg/mL puromycin (Sigma) for 2-4 days in 6-well plates. The target sequences of shRNA oligonucleotides were listed in “Supplementary table 2”.

**The generation of stable knockout cell lines**

HeLa and HCT116 gene knockout cell lines were all designed from website crispr.mit.edu, and generated by using CRISPR/Cas9 gene editing. Briefly, gene targeting DNA fragments specific for human Yme1L, OPA1, Mic19, Mic10, or ATAD3A DNA was synthesized, and subcloned into LentiCRISPR plasmid (Addgene, deposited by Feng Zhang). The target sequences of knockout oligonucleotides were listed in “Supplementary table 2”. The respective plasmids were transfected in 293T cells together with plasmids psPAX2 and VSV-G using Lipofectamine 2000 (Invitrogen). After two days of transfection, the medium containing the target lentiviral particles were collected to infect HeLa and HCT116 cells. The infected cells were selected by puromycin (2 μg/mL), then the single cells were sorted and cultured in 96-well dishes. Two weeks later, monoclonal cells were screened and identified by Western blotting using the specific antibodies.

**FEI Tecnai Spirit microscope analysis**

HCT116 OPA1 KO cells were embedded in EPON 812 resin, 70nm sections. Single axis tilt series of mitochondria (+/-60°, step size 1.5°) were collected using an FEI Tecnai Spirit microscope operating at 80 KV, equipped with 2000 x 2000 FEI Eagle CCD camera. Images were acquired with a specimen pixel size of 6 Å and a defocus of 5-8 µm. The total accumulated electron dose was limited to 120 electrons/Å2 per tilt series. Tilt series were aligned using cellular organelle contours as fiducials, and 3D tomographic volumes were reconstructed using the IMOD software.

**Mitochondrial membrane potential measurement**

HeLa Cells in 6-well plates were collected and washed with PBS, and then cells were incubated at 37°C under 5% CO2 with 250nM TMRM in DMEM without FBS for 30 minutes. Cells washed three times and analyzed by FACS.

**REFERENCE**

1. Chen H, Chomyn A, Chan DC. Disruption of fusion results in mitochondrial heterogeneity and dysfunction. *The Journal of biological chemistry* 2005, **280**(28)**:** 26185-26192.

**SUPPLEMENTARY VIDEOS INFORMATION**

**Supplementary Video 1**

Live HeLa cells were stained with Mitotracker Green (250nM, 15min), and imaged under 10ms exposure with an initial illumination intensity of ~18 W/cm2 light intensity (which increased by 0.05% during each SIM image). Each SIM frame was acquired over 100ms, a raw data exposure time of 10ms.

**Supplementary Video 2**

Live WT MEFs were stained with Mitotracker Green (250nM, 15min), and imaged by Hessian-SIM. Each SIM frame was acquired over 100ms, a raw data exposure time of 10ms.

**Supplementary Video 3**

Live OPA1 KO MEFs were stained with Mitotracker Green (250nM, 15min), and imaged by Hessian-SIM. Each SIM frame was acquired over 100ms, a raw data exposure time of 10ms.

**Supplementary Video 4**

Live HCT116 cells were stained with Mitotracker Green (250nM, 15min), and imaged by Hessian-SIM. Each SIM frame was acquired over 100ms, a raw data exposure time of 10ms.

**Supplementary Video 5**

Live HCT116 Yme1L KO cells were stained with Mitotracker Green (250nM, 15min), and imaged by Hessian-SIM. Each SIM frame was acquired over 100ms, a raw data exposure time of 10ms.

**Supplementary Video 6**

Live HeLa Mic10 KO cells were stained with Mitotracker Green (250nM, 15min), and imaged by Hessian-SIM. Each SIM frame was acquired over 100ms, a raw data exposure time of 10ms.

**Supplementary Video 7**

Live HeLa Mic19 KO cells were stained with Mitotracker Green (250nM, 15min), and imaged by Hessian-SIM. Each SIM frame was acquired over 100ms, a raw data exposure time of 10ms.

**Supplementary Video 8**

Live HeLa Mic60 knockdown (KD) cells were stained with Mitotracker Green (250nM, 15min), and imaged by Hessian-SIM. Each SIM frame was acquired over 100ms, a raw data exposure time of 10ms.

**Supplementary Video 9**

Live HeLa Sam50 KD cells were stained with Mitotracker Green (250nM, 15min), and imaged by Hessian-SIM. Each SIM frame was acquired over 100ms, a raw data exposure time of 10ms.

**Supplementary Video 10**

Live HeLa ATAD3A KO cells were stained with Mitotracker Green (250nM, 15min), and imaged by Hessian-SIM. Each SIM frame was acquired over 100ms, a raw data exposure time of 10ms.

**Supplementary Video 11**

Focused ion beam/scanning electron microscopy (FIB-SEM) FIB-SEM recording of an HeLa mitochondria. Displayed is an orthoslice moving through the data stack recorded by FIB-SEM, the arrow indicates the mitochondrion containing the “cut-through crista”. Then, FIB-SEM images of several segments of a mitochondrion from HeLa cells were used for 3D reconstruction by the 3D IMOD software. The outer membrane (OMM, white), inner boundary membrane (IBM, cyan), and crista membrane (Purple red).

**Supplementary Video 12**

FIB-SEM recording of an HCT116 OPA1 KO mitochondria. Displayed is an orthoslice moving through the data stack recorded by FIB-SEM. Then, FIB-SEM images of several segments of a mitochondrion from HCT116 OPA1 KO cell were used for 3D reconstruction by the 3D IMOD software. The outer membrane (OMM, white), inner boundary membrane (IBM, cyan), and crista membrane (purple-red).

**Supplementary Video 13**

FIB-SEM recording of a HeLa Mic10 KO mitochondria. Displayed is an orthoslice moving through the data stack recorded by FIB-SEM, the arrow indicates the mitochondrion containing the crista lacking crista junctions. Then, 3D reconstruction of FIB-SEM images of several segments of a mitochondrion from HeLa Mic10 KO cell were performed using the 3D IMOD software. The outer membrane (OMM, white), inner boundary membrane (IBM, cyan), and crista membrane (purple-red and yellow).

**Supplementary Video 1**4

FIB-SEM recording of a HeLa Mic10 KO mitochondria containing the spherical crista. Displayed is an orthoslice moving through the data stack recorded by FIB-SEM, the arrow indicates the mitochondrion containing the spherical crista. Then, 3D reconstruction of FIB-SEM images of several segments of a mitochondrion containing the spherical crista from HeLa Mic10 KO cell were performed using 3D IMOD software. The outer membrane (OMM, white), inner boundary membrane (IBM, cyan), and crista membrane (purple-red and yellow).

**Supplementary Video 15**

FIB-SEM recording of a HCT116 OPA1 KO mitochondria. Displayed is an orthoslice moving through the data stack recorded by FIB-SEM, the arrow indicates the mitochondrion containing the spherical cristae. Then, FIB-SEM images of several segments of a mitochondrion from HCT116 OPA1 KO cell were used for 3D reconstruction by the 3D IMOD software. The outer membrane (OMM, white), inner boundary membrane (IBM, cyan), and crista membrane (purple-red).

**Supplementary Video 16**

FIB-SEM recording of HCT116 Mic10-OPA1 DKO mitochondria (Representative mitochondrion 1, RM1). Displayed is an orthoslice moving through the data stack recorded by FIB-SEM, the arrow indicates the mitochondrion containing the spherical crista. Then, 3D reconstruction of FIB-SEM images of several segments of a mitochondrion (Representative mitochondrion-1, RM1) from HCT116 Mic10-OPA1 DKO cell were performed using the 3D IMOD software. The outer membrane (OMM, white), inner boundary membrane (IBM, cyan), and spherical crista membrane (purple-red and yellow).

**Supplementary Video 17**

FIB-SEM recording of HCT116 Mic10-OPA1 DKO mitochondria (Representative mitochondrion-2 and -3, RM2 and RM3). Displayed is an orthoslice moving through the data stack recorded by FIB-SEM, the arrowheads indicate the mitochondrion containing spherical crista, the upper arrowhead indicates “mitochondrion R2” and the below arrowhead indicates “mitochondrion R3”.

**Supplementary Videos 18-19**

3D reconstruction of FIB-SEM images of several segments of two mitochondria (Representative mitochondrion-2 and -3, RM2 and RM3) from HCT116 Mic10-OPA1 DKO cell using the 3D IMOD software. The outer membrane (OMM, white), inner boundary membrane (IBM, cyan), and spherical crista membrane (purple-red).

**Supplementary Video 20**

Live COS-7 Mic10 KO cells were stained with Mitotracker Green (250nM, 15min), and imaged by Hessian-SIM. Each SIM frame was acquired over 100ms, a raw data exposure time of 10ms.

**Supplementary Video 21**

Live COS-7 Mic10 KO cells were stained with Mitotracker Green (250nM, 15min), and imaged by Hessian-SIM. Each SIM frame was acquired over 100ms, a raw data exposure time of 10ms. For the time-lapse images, the time interval between 2 SIM images was set to 500ms.

**SUPPLEMENTARY TABLES**

**Supplementary Table 1.** **Selected Tandem mass spectrometry (MS/MS) data from the eluted protein samples of the co-IP assay.**

| **Protein Name** | **Description** | **MW (kDa)** | **Σ# Unique Peptides** | **Σ# PSMs** |
| --- | --- | --- | --- | --- |
| Mic60/IMMT | Mitochondrial inner membrane protein | 83.63 | 44 | 75 |
| Mic19/CHCHD3 | Coiled-coil-helix-coiled-coil-helix domain-containing protein 3 | 26.14 | 14 | 17 |
| ATAD3A | ATPase family AAA domain-containing protein 3A | 71.325 | 18 | 25 |
| MTX2 | Metaxin 2 | 32.87 | 5 | 7 |
| Mic25/CHCHD6 | Coiled-coil-helix-coiled-coil-helix domain-containing protein 6 | 26.44 | 3 | 3 |
| ATAD3B | ATPase family AAA domain-containing protein 3B | 72.527 | 15 | 20 |
| ZNHIT2 | Zinc finger HIT domain-containing protein 2 | 42.86 | 2 | 2 |
| HSPD1 | 60kDa heat shock protein, mitochondrial | 61.02 | 8 | 9 |
| PGAM5 | Serine/threonine-protein phosphatase PGAM5 | 31.98 | 8 | 11 |
| ATP5B | ATP synthase subunit beta, mitochondrial | 56.52 | 3 | 3 |
| SLC25A13 | Calcium-binding mitochondrial carrier protein Aralar2 | 74.13 | 5 | 6 |
| COX6C | Cytochrome c oxidase subunit 6C | 8.77 | 1 | 1 |
| UQCRC2 | Cytochrome b-c1 complex subunit 2, mitochondrial | 48.41 | 9 | 10 |

**Supplementary Table 2**

**List of primers for the construction of shRNAi plasmids and knockout plasmids.**

| **Target gene** | **Oligonucleotides sequence** |
| --- | --- |
| Homo Sam50 | F5’-GATCCCCGGTCATCGATTCTCGGAATTTCAAGAGAATTCCGAGAATCGATGACCTTTTTGGAAA-3’  R5’-AGCTTTTCCAAAAAGGTCATCGATTCTCGGAATTCTCTTGAAATTCCGAGAATCGATGACCGGG-3’ |
| Homo Mic60 | F5’-GATCCCCGCCCGAATGACTCTAGAAATTCAAGAGATTTCTAGGGTCATTCGGGCTTTTTGGAAA-3’  R5’-AGCTTTTCCAAAAAGCCCGAATGACCCTAGAAATCTCTTGAATTTCTAGGGTCATTCGGGCGGG-3’ |
| Homo Mic10 | F 5’- CACCTGTCTGAGTCGGAGCTCGGC -3’  R 5’- AAACTGTCTGAGTCGGAGCTCGGC -3’ |
| Homo Mic19 | F 5’-CACCTCGGGAGAGGATATGTAGCG-3’  R 5’-AAACTCGGGAGAGGATATGTAGCG-3’ |
| Homo ATAD3A | F 5’-CACCGAATGAGATGCTGCGAGTGG-3’  R 5’-AAACGAATGAGATGCTGCGAGTGG-3’ |
| Homo Yme1L | F 5’- CACCTGTCCAAGTGTTGGCCCCCG -3’  R 5’-AAACTGTCCAAGTGTTGGCCCCCG-3’ |
| Homo OPA1 | F 5'-CACCGCGACTACGTCGGGCCGCTG-3'  R 5'-AAACCAGCGGCCCGACGTAGTCGC-3' |
